# Macrophage Tim-4 protects against deep vein thrombosis by binding CK2β to suppress inflammatory responses

**DOI:** 10.3389/fimmu.2025.1634230

**Published:** 2025-09-23

**Authors:** Xiao Wang, Zhen Zhang, Weiwei Zheng, Baohui Zhang, Chu Chu, Junjie Chen, Cui Liu, Ke Xu, Zhijun Yu, Qiang Guo, Songbo Zhao, Xu Chen, Fuxiang Bai, Bin Wang, Xia Li, Wen Liu

**Affiliations:** ^1^ Department of Central Laboratory, Shandong Provincial Hospital Affiliated to Shandong First Medical University, Jinan, Shandong, China; ^2^ Department of Immunology, School of Clinical and Basic Medical Sciences, Shandong First Medical University and Shandong Academy of Medical Sciences, Jinan, Shandong, China; ^3^ Innovative Institute of Chinese Medicine and Pharmacy, Shandong University of Traditional Chinese Medicine, Jinan, Shandong, China; ^4^ Department of Orthopedic, Qilu Hospital of Shandong University, Jinan, Shandong, China; ^5^ Biomedical Sciences College & Shandong Medicinal Biotechnology Centre, Shandong First Medical University and Shandong Academy of Medical Sciences, Jinan, Shandong, China; ^6^ Hospital for Skin Diseases, Shandong First Medical University, Jinan, Shandong, China; ^7^ The Second Affiliated Hospital of Shandong University of Traditional Chinese Medicine, Jinan, Shandong, China

**Keywords:** Tim-4, deep vein thrombosis, Ck2β, lncRNF219-3:1, miR-93-5p

## Abstract

**Background:**

Deep vein thrombosis (DVT) is a venous reflux disorder caused by dysregulated coagulation, with macrophage inflammatory responses being critical for its progression. T-cell immunoglobulin and mucin domain containing 4 (Tim-4) is known as a key regulator of macrophage function and inflammation. However, its involvement in DVT remains completely unclear.

**Methods:**

Tim-4 expression was comprehensively assessed in peripheral blood mononuclear cells from DVT patients and inferior vena cava-associated macrophages in both clinical specimens and murine DVT models using integrated approaches including single-cell RNA sequencing, immunofluorescence, flow cytometry, quantitative PCR, and Western blot. Macrophage-specific *Tim-4* knockout mice (LysM-Cre; *Tim-4* ^fl/fl^) and littermate controls (*Tim-4* ^fl/fl^) were constructed to investigate the role of macrophage Tim-4 in DVT. The interaction between Tim-4 and CK2β was verified by mass spectrometry and co-immunoprecipitation. The lncRNF219-3:1/miR-93-5p/Tim-4 regulatory axis was validated through RNA pull-down, RNA antisense purification, and luciferase reporter assays.

**Results:**

Tim-4 was significantly downregulated in DVT-associated macrophages, correlating with elevated proinflammatory cytokine levels. Macrophage-specific *Tim-4* knockout aggravated DVT progression both *in vitro* and *in vivo*. Mechanistically, Tim-4 directly bound casein kinase 2β (CK2β) regulatory subunit, suppressing CK2 holoenzyme activity and subsequent NF-κB pathway activation (pP65 and pIκBα). Notably, pharmacologically blocking CK2 activation or the NF-κB pathway abolished the pro-thrombotic effects of *Tim-4* deficiency. Furthermore, we identified a novel ceRNA network wherein lncRNF219-3:1 acted as a miR-93-5p sponge to indirectly upregulate Tim-4 expression, thereby enhancing anti-inflammatory macrophage responses and attenuating thrombus formation.

**Conclusions:**

Our findings demonstrate that macrophage Tim-4, regulated by lncRNF219-3:1/miR-93-5p axis, functions as a critical suppressor of DVT through hijacking and sequestering CK2β to dampen NF-κB mediated inflammation. The study unveils novel immunomodulatory mechanisms in DVT pathogenesis and highlights Tim-4 and its regulatory network as potential therapeutic targets for DVT in clinic.

## 1 Introduction

Deep vein thrombosis (DVT) is caused by abnormal coagulation of blood in deep veins. If the thrombus dislodges from the vein wall, it can travel to and deposit in the pulmonary vessels, block blood circulation, and form a pulmonary embolism (1). Together, these conditions are referred to as venous thromboembolism (VTE), which has become the third leading vascular disease after acute myocardial infarction and stroke (2). VTE is a serious vascular disease with an estimated annual incidence rate of one in a thousand, and two-thirds of these cases are DVT (3). The pathophysiological mechanisms leading to thrombosis are traditionally explained by Virchow’s triad: blood flow disturbance, endothelial injury, and hypercoagulability (4). Although a wide range of anticoagulant therapies exist, DVT-related cardiovascular events remain widespread due to major side effects such as allergies and bleeding (5). Therefore, it is urgent to explore the pathogenesis of DVT to find more effective treatment methods or drug targets. Investigating the pathophysiology of DVT is imperative for developing more potent therapeutic targets and treatment approaches.

Macrophage-mediated inflammatory responses are critical steps in the progression of DVT, as demonstrated by monocyte-derived macrophages from DVT patients accelerating thrombosis by elevated expression of endothelial adhesion molecules, including E-selectin, vascular cell adhesion molecule 1 (VCAM-1), and intercellular adhesion molecule 1 (ICAM-1) (6). Moreover, activated macrophages exacerbate inflammation-mediated thrombosis by secreting tumor necrosis factor (TNF)-α, interleukin (IL)-1β, and IL-6, which directly induce tissue factor (TF) production and von Willebrand factor (vWF)-dependent platelet adhesion during early DVT pathogenesis (7–9). Notably, IL-6 enhances thrombocytosis via hepatic thrombopoietin induction, while IL-1β directly activates platelet integrins (10, 11). These findings highlight the therapeutic potential of neutralizing monoclonal antibodies targeting IL-6 or IL-1β in cardiovascular diseases (12, 13). As an important pathway mediating inflammatory responses in macrophages, NF-κB signaling is also a key bridge connecting macrophage-mediated inflammation and thrombosis (14). Its activation is modulated by a set of molecules including kinases, phosphatases, and ubiquitin-modifying enzymes. Protein kinase CK2 (formerly casein kinase II or 2) is a highly conserved heterotetrameric complex composed of a central dimer of two regulatory β subunits (CK2β) that binds two α and/or α’ catalytic subunits (CK2α) structurally (15). Functionally, CK2 is a multifunctional, ubiquitously expressed, and constitutively active protein kinase that phosphorylates a set of substrates involved in various biological processes, including cell proliferation, differentiation and the DNA damage responses, and implicates in many human malignancies and other disorders (16). Previous evidence indicated that CK2 could mediate aberrant activation of NF-κB by promoting phosphorylation of IκBα, enhancing IκBα proteasomal degradation, and also directly phosphorylating P65 to finally increased NF-κB transcriptional activity (17–19). Collectively, these data indicate that targeting CK2 dependent NF-κB activation in macrophages may be a promising therapeutic strategy to reverse DVT. Nevertheless, the executioners or initiators of CK2/NF-κB activation-mediated inflammatory response imbalances in DVT are still unclear. Further exploration of the pathogenesis of macrophage inflammatory responses in DVT development and key regulators are essential for preventing and treating DVT.

The gene family of T-cell immunoglobulin and mucin domain (Tim) was identified in asthma and allergic mouse models through targeted cloning in 2001 (20). The Tim gene family includes eight members (Tim-1 to Tim-8) on mouse chromosome 11B1.1 and three members (Tim-1, Tim-3, and Tim-4) on human chromosome 5q33.2, regions which were related to asthma, allergy, and autoimmune reactions (21). Furthermore, Tim-1, Tim-3, and Tim-4 are highly homologous in humans and mice (22). Tim-4, a member of the Tim family, is highly expressed in macrophages and activated dendritic cells, but not in T cells (21). Identified as a phosphatidylserine (PS) receptor, Tim-4 enhances the phagocytic activity of macrophages by binding with PS exposed on the surface of apoptotic cells and regulates the secretion of cytokines associated with macrophages (23). Macrophage Tim-4 has been reported to either suppress (a predominant role) or enhance inflammatory responses, playing context-dependent roles in various disease pathologies. Transfer of Tim-4 overexpressing macrophages can inhibit the production of inflammatory cytokines TNF-α and IL-6 in macrophages, thereby significantly alleviating ConA-induced liver inflammatory injury and suppressing the occurrence of LPS-induced sepsis (24, 25). In patients with type 2 diabetes, up-regulation of macrophage Tim-4 expression can inhibit macrophage activation-mediated inflammatory states to maintain cellular homeostasis (26). By activating the autophagy pathway, macrophage Tim-4 promotes NLRP3 inflammasome degradation and consequently diminishes inflammatory damage to the liver (27). A recent study observed that *Tim-4* knockout promoted M1-like macrophage polarization *via* suppression of TLR4 internalization to increase the secretion of inflammatory factors in adipose tissue (28). However, whether Tim-4 is involved in inflammatory responses related to DVT and underlying mechanisms remains unclear.

Non-coding RNAs (ncRNAs) generally refer to a set of RNA molecules encoded by the genome that are not translated into proteins, mainly including microRNAs (miRNAs), long non-coding RNAs (lncRNAs), and circular RNAs, which are involved in transcriptional regulation (29, 30). miRNAs with a length of about 22 nucleotides (nt) are a well-characterized class of ncRNAs and usually play a vital role as post-transcriptional regulators of gene expression (31). miRNAs can bind to the 3’-untranslated region (3’UTR) of target gene mRNAs through base complementary pairing to inhibit the translation or degrade target genes, thereby reversely regulating the expression of target genes and participating in the progression of various diseases (32). After miRNAs, lncRNAs were identified as a distinct class of linear transcripts exceeding 200nt in length (33). LncRNAs, rich in sequences complementary to miRNAs, are called competing endogenous RNAs (ceRNAs) that function as molecular sponges for miRNAs (34, 35). By competitively binding and blocking the complementary binding sites between miRNAs and target genes, lncRNAs can up-regulate the expression of target genes by relieving the suppression of miRNAs on target genes, and indirectly participate in the occurrence and development of atherosclerosis, hypertension, and other vascular diseases (36, 37). Several reports have shown that the abnormal expression of ceRNAs is involved in the progression of DVT. Sun et al. reported that up-regulation of lncRNA GUSBP5-AS in endothelial progenitor cells (EPCs) of DVT patients regulated the expression of FGF2 and MMP2/9 through the miR-223-3p/FOXO1/Akt pathway, promoted the homing ability of EPCs to the thrombus site, and facilitated the recanalization of the thrombus (38). Zhu et al. showed that LINC00659 could promote the apoptosis of vascular endothelial cells by regulating the miR-525-5p/Bax axis, thus accelerating the development of DVT (39). Therefore, as miRNAs sponges, lncRNAs play an important role in the progression of DVT by regulating the expression of target genes. In addition, a recent study has found that lncRNA NEAT1, as a ceRNA of miR-202-3p, up-regulates the expression of Tim-4 and promotes the malignant metastasis of endometrial cancer (40). However, the potential involvement of ceRNA networks in regulating Tim-4-mediated macrophage inflammatory responses during DVT progression remains unexplored.

In this study, we aimed to elucidate the role of macrophage Tim-4 in the pathogenesis of DVT, with particular emphasis on its potential crosstalk with inflammatory signaling pathways. Specifically, we sought to determine whether macrophage-specific *Tim-4* deficiency exacerbates DVT via proinflammatory cytokine activation, how Tim-4 mechanistically modulates the CK2β/NF-κB axis in macrophages, and whether upstream epigenetic regulators drive macrophage Tim-4 downregulation during DVT progression. By addressing these questions, our study identifies novel therapeutic targets for DVT intervention within lncRNF-219-3:1/miR-93-5p regulated Tim-4/CK2β/NF-κB pathway, thereby bridging the gap between epigenetic regulation and innate immune responses in DVT development.

## 2 Materials and methods

### 2.1 Mice

*Tim-4* floxed (*Tim-4* ^fl/fl^) C57BL/6J mice were generated from Cyagen Corporation (Suzhou, China) by CRISPR/Cas-mediated genome engineering. Briefly, a *Tim-4* floxed targeting vector, containing the mouse *Tim-4* gene, spanning the region from intron 1 to intron 3, and two loxP sites, was constructed. Then the targeting vector, Cas9, and gRNA were co-injected into fertilized eggs for *Tim-4* ^fl/fl^ mouse production. Macrophage-specific *Tim-4* knockout mice (LysM-Cre; *Tim-4* ^fl/fl^) were generated by crossing LysM-Cre and *Tim-4* ^fl/fl^ mice. *Tim-4* ^fl/fl^ and LysM-Cre; *Tim-4* ^fl/fl^ mice were reared and propagated in the Experimental Animal Center of Shandong First Medical University. All animals were housed in a specific pathogen-free facility that maintained a 12 hours light and 12 hours dark cycle, and had free access to food and water. The 6-8-week-old male mice were used in all experiments, and the mice were divided into experimental groups at random.

### 2.2 Patient samples

Randomly collect fresh peripheral venous blood specimens, as well as lower limb venous thrombotic tissue and control venous vascular tissue from healthy individuals and DVT patients at the Affiliated Hospital of Shandong University of Traditional Chinese Medicine and Qilu Hospital of Shandong University. PBMCs were isolated by Ficoll density-gradient centrifugation, then total RNA was extracted and detected by quantitative PCR (qPCR). DVT was confirmed by both color doppler ultrasound and lower extremity angiography.

### 2.3 Establishment of DVT model mice

The IVCs of the mice were ligated to establish a DVT model. Briefly, the mice were anesthetized by intraperitoneal injection of tribromoethanol (0.2 ml/10 g body weight; M2920, AibeiBio, Nanjing, China), and they were fixed on the operating table in a supine position. The abdomen was shaved with a shaving machine and disinfected with 0.5% povidone iodine solution. Then, the abdominal skin was cut open to expose IVCs, the operation line was ligated, and the skin of mice was finally sutured. After the mice regained consciousness, they were fed a normal diet. In the NF-κB inhibitor group, mice with the indicated genotype were injected intraperitoneally with a dose of 4 mg/kg BAY11-7082 (abs810013, Absin, Shanghai, China), an NF-κB inhibitor delivered 1 hour prior to DVT modeling. In the CK2 inhibitor group, mice were treated with CX-4945 (A11060, AdooQ Bioscience, Irvine, CA) by intraperitoneal injection at a dose of 40 μg/g after DVT modeling. At the end of the experiment, the mice were euthanized after inhaling 100% CO2 and undergoing cervical dislocation.

### 2.4 Primary macrophage isolation and activation

Peritoneal macrophages (PEMs) or bone marrow-derived macrophages (BMDMs) were isolated from the peritoneal cavity and the femur and tibia from mice with the indicated genotypes, respectively. Mice were intraperitoneally injected with 1 ml of sterile 6% starch solution, and PEMs were harvested with DMEM (C3113-0500, VivaCell, Shanghai, China) after 3 days of injection. The tibia and femur of mice were isolated, and bone marrow cells were flushed with a 1 ml sterile syringe and BMDMs were differentiated with 100 ng/ml M-CSF (abs04383, Absin, Shanghai, China) for about 6 days. The isolated PEMs and BMDMs were cultured in DMEM, adding 10% fetal bovine serum (FBS, 04-001-1A, Biological Industries, Israel) and 1% penicillin-streptomycin (P1400, Solarbio, Beijing, China). To induce macrophage activation, PEMs or BMDMs were incubated with 1 μg/ml lipopolysaccharide (LPS) for 3.5 hours.

### 2.5 Cell culture

All cells were cultured at 37°C with 5% CO_2_ and the corresponding medium. The human macrophage cell line THP-1 was cultured in RPMI 1640 medium (C3010-0500, VivaCell, Shanghai, China) plus 10% FBS. The human umbilical vein endothelial cells (HUVECs) used in the study were cultured in DMEM/F-12 (1:1) medium (C11330500BT, Gibco, China) containing 10% FBS. Above cell lines were cultured with penicillin-streptomycin (100 U/ml) solution.

### 2.6 Real-time qPCR analysis

Total RNA was extracted from cultured cells and vascular tissues with TRIzol reagent (DP424, TIANGEN, China). Then, total RNA (1 μg) was used to synthesize complementary DNA using the ReverTra Ace qPCR RT kit (FSQ-101, TOYOBO, Japan). The expression of Tim-4, IL-1β, TNF-α, miR-93-5p, and lncRNF219-3:1 was detected by qPCR using SYBR Green PCR reagent (PC6202, Aidlab, Beijing, China) and the LightCycler 96 real-time fluorescence qPCR system (Roche).

### 2.7 Western blot analysis

Tissues or cells were incubated on ice for 30 minutes in RIPA lysis buffer (R0010, Solarbio, Beijing, China) containing 1% protease inhibitors (P0100, Solarbio, Beijing, China) and phosphatase inhibitors (00-4980-93, Invitrogen, USA). The samples were centrifuged at 4°C and 12000 rpm for 5 minutes, and the supernatant was collected. The protein solution was mixed with 5 × loading buffer and heated at 100°C for 7 minutes to denature the protein. Approximately 50 μg protein extract was fractionated by a 10% SDS-PAGE gel and then transferred onto a PVDF membrane (IPVH00010, Millipore, Darmstadt, Germany). The membrane was blocked with 5% bovine serum albumin (BSA) (A8020, Solarbio, Beijing, China) at room temperature for 2 hours and then incubated with the appropriate primary antibodies against Tim-4 (12008-1-AP, Proteintech, Wuhan, China), CK2β (GB113852-50, Servicebio, Wuhan, China), TF (CY5807, Abways, Shanghai, China), vWF (11778-1-AP, Proteintech, Wuhan, China), P65 (CY5034, Abways, Shanghai, China), pP65 (3033S, Cell Signaling Technology, Danvers, MA, USA), IκBα (10268-1-AP, Proteintech, Wuhan, China), pIκBα (82349-1-RR, Proteintech, Wuhan, China), and GAPDH (AB0037, Abways, Shanghai, China) overnight at 4°C. After being washed for 3 times in PBST (PBS and Tween-20) washing buffer, the membranes were incubated with anti-rabbit (GAR0072, MULTI SCIENCES, Hangzhou, China) or anti-mouse horseradish peroxidase (HRP)-labeled secondary antibody (GAM0072, MULTI SCIENCES, Hangzhou, China) at room temperature for 1.5 hours. Protein bands were visualized using Immobilon^®^ Western Chemiluminescent HRP Substrate (WBKLS0500, Millipore, Darmstadt, Germany) in the ChemiDoc M system.

### 2.8 Immunofluorescence staining

Freshly obtained thrombus samples were fixed in 4% paraformaldehyde (BL539A, Biosharp, Hefei, China) for 24 hours, followed by standard paraffin embedding and sectioning at 4 μm thickness. Antigen retrieval was performed using EDTA Antigen Retrieval Solution (pH 9.0, ZLI-9068, ZSGB-BIO, Beijing, China) at 95°C for 20 minutes. Endogenous peroxidase blockers were added to the slides and incubated at 37°C for 15 minutes. After blocking, sections were washed three times in PBST (PBS + 0.1% Tween-20, pH 7.4), 5 minutes per wash with gentle agitation at room temperature. Normal goat serum was added to the slides to further block and incubated at 37°C for 30 minutes. Then, appropriate primary antibodies against CD68 (66231-2-Ig, Proteintech, Wuhan, China), F4/80 (ab6640, Abcam, Cambridge, UK), Tim-4 (sc-390805, Santa Cruz Biotechnology, Shanghai, China; 12008-1-AP, Proteintech, Wuhan, China), and CK2β (GB113852-50, Servicebio, Wuhan, China) were incubated overnight at 4°C. The sections were then washed 3 × 5 minutes in PBST and incubated with corresponding secondary antibodies (Alexa Fluor^®^ 594-conjugated antibodies, ZF-0513, ZF-0516; FITC/Alexa Fluor^®^ 488-conjugated antibodies, ZF-0312, ZF-0511, ZSGB-BIO, Beijing, China). Nuclei were counterstained with DAPI (10 μg/ml, C1002, Beyotime, Shanghai, China) for 5 minutes. Images were acquired using a conventional inverted microscope camera system (Olympus) and a LSM 780 confocal microscope (Carl Zeiss).

### 2.9 Flow cytometry analysis

FCM was used to analyze the expression of Tim-4 in PEMs, BMDMs, and spleen mononuclear cells. During the staining process, the cells were incubated with antibodies at 4°C for 30 minutes in the dark and then washed twice with 2 ml of PBS (P1020, Solarbio, Beijing, China). After centrifugation, supernatant was discarded, and 200 μl of PBS was added to re-suspend the cells for detection. The information of antibodies are as follows: PE/Cy7-conjugated anti-mouse Tim-4 (130010, Biolegend, USA); APC-conjugated anti-mouse F4/80 (17-4801-82, Invitrogen, USA); Alexa Fluor 488-conjugated anti-mouse CD11b (53-0112-82, Invitrogen, USA); PerCP/Cyanine5.5-conjugated anti-mouse Ly6C (128012, BioLegend, USA); PE/Dazzle (ECD) -conjugated anti-mouse Ly6G (127648, BioLegend, USA); PE-conjugated anti-mouse Tim-4 (130006, Biolegend, USA). Data were acquired using the BD flow cytometer and analyzed using flowjo.

### 2.10 Rigid protein–protein docking (ZDOCK)

ZDOCK was performed between Tim-4 and CK2β to predict their binding interactions. The Protein Data Bank format of the protein structural domain was downloaded from the PDB database (http://www.rcsb.org/). The ZDOCK module was run to identify the docking sites and calculate the ZDOCK scores.

### 2.11 Co-immunoprecipitation and liquid chromatography-tandem mass spectrometry

Immunoprecipitation (IP) was performed using an anti-Tim-4 antibody (IP: Tim-4) or IgG (IP: IgG) in PEMs, and the protein lysates were used for LC-MS/MS (Novogene, Beijing, China). The protein complexes, pulled down by Co-IP, were assessed by 12% SDS-PAGE. The protein gel was sliced and digested with trypsin. The process was separated by high performance liquid chromatography and analyzed by tandem mass spectrometry. With the help of the retrieval software and the corresponding protein database, the collected mass spectrometry data could be analyzed to determine the protein composition of the sample. Co-IP validation of binding between Tim-4 and CK2β was also performed using antibodies against Tim-4 (sc-390805, Santa Cruz Biotechnology, Texas, USA), HA (GB151252-100, Servicebio, Wuhan, China), Flag (GB15939-100, Servicebio, Wuhan, China), or IgG (sc-2025, Santa Cruz Biotechnology, Texas, USA) in PEMs and HEK293T cells. Immunoblotting with anti-Tim-4, anti-CK2β, anti-HA, and anti-Flag antibodies was performed.

### 2.12 Single-cell RNA Sequencing dataset processing and annotation

Single-cell RNA sequencing dataset (GSE221978) was processed using Seurat (v.4.0.3) in R following established computational pipelines. Raw gene expression matrices were imported *via* the Read10X_h5 function and subjected to rigorous quality control by assessed three key metrics: nFeature_RNA (number of detected genes per cell), nCount_RNA (total RNA counts per cell), and percent.mt (mitochondrial gene proportion). Cells with percent.mt < 20%, 1000 < nFeature_RNA < 9000, nCount_RNA < 100000, were retained. After preprocessing, we performed downstream analysis, including: Highly Variable Gene selection, data normalization (log-normalization), and principal component analysis-based dimensionality reduction. Batch correction was conducted using MAESTRO (v1.3.0). Finally, Cell types were annotated using SingleR (v1.10.0) with the ImmGen database (Immunological Genome Project) by comparing gene expression profiles between test data and reference datasets, with assigned labels stored in the Seurat object metadata. The distribution patterns and expression differences of target genes across distinct cell populations were then visualized using t-SNE dimensionality reduction.

### 2.13 Statistical analysis

All data were expressed as the mean ± standard deviation (SD) of at least three independent experiments. GraphPad Prism 10.0 software was used for data analysis. Unpaired data conforming to both normal distribution and homogeneity of variance between two independent samples were compared using unpaired t-test, and one-way analysis of variance (ANOVA) along with Tukey’s *post-hoc* multiple comparisons test were employed for comparing more than two groups. If the data satisfied normal distribution but not homogeneity of variance, the unpaired t-test with Welch’s correction was used for comparisons between two groups, and Welch’s ANOVA test for multiple groups. If data for normality test failed, nonparametric Mann-Whitney test was used for comparisons between two groups, and the Fired man or Kruskal-Wallis test for comparisons among multiple groups. *p* < 0.05 represents a statistically significant difference.

Additional detailed Materials and Methods are provided in Section 2 of **Supplementary Material 1** (**Supplementary Material** and **Supplementary Method**).

## 3 Results

### 3.1 Tim-4 is significantly decreased in PBMCs from DVT patients and macrophages from IVCs of DVT mice, while pro-inflammatory cytokines are elevated

To explore the role of Tim-4 in DVT, we firstly analyzed Tim-4 mRNA expression level from our previous high-throughput whole transcriptome sequencing results and found that the expression of Tim-4 was down-regulated, while the expression of pro-inflammatory mediators was up-regulated in PBMCs of DVT patients (**Supplementary Figures S1A, B**). Besides, the top 20 KEGG-enriched signaling pathways analysis revealed that the NF-κB signaling pathway was the most prominently enriched pathway in the PBMCs of DVT patients (**Supplementary Figure S1C**). Then we further detected Tim-4 expression in IVCs from successfully established DVT mice *in vivo* for indicated time points (**Supplementary Figures S2A, B**). The mRNA and protein levels of Tim-4 in IVCs displayed a tendency of first declining and then tended to raising over time by qPCR and western blot analysis, which were contrary to the tendency of the pro-thrombotic mediators (vWF and TF) expression at translational level, as well as with P65 phosphorylation-a key regulator of NF-κB pathway activation (**Supplementary Figures S2C, D**). In contrast, mRNA levels of pro-inflammatory genes (IL-1β and TNF-α) exhibited a “rise-decline-rise” pattern in **Supplementary Figure S2C**. In accordance, immunofluorescence (IF) staining results further confirmed the same tendency of Tim-4 expression in IVCs from DVT mice (**Supplementary Figure S2E**). The above findings suggested that decreased Tim-4 in IVCs from DVT mice might be associated with the upregulated expression of pro-inflammatory factors and thrombus formation.

**Figure 1:**
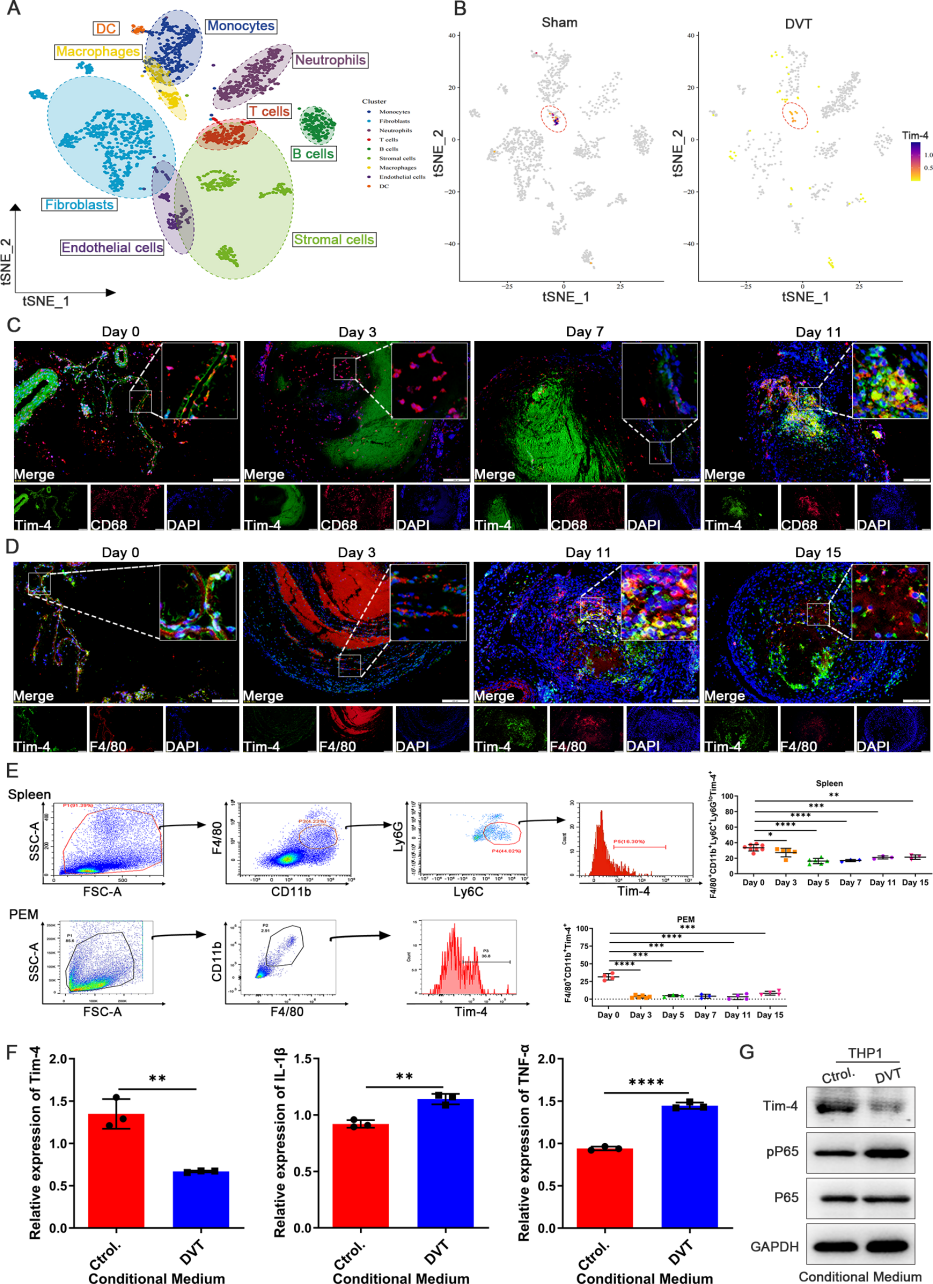
Tim-4 expression in macrophages is first down-regulated and subsequently up-regulated in DVT mice. **(A)** A vein wall cell atlas of murine venous thrombosis and sham group determined by single-cell RNA sequencing (scRNA-seq) based on bioinformatics of GSE221978 dataset. **(B)** Tim-4 expression in different cell clusters from sham and DVT mice was displayed by cell atlas. **(C, D)** Representative IF photomicrographs of co-staining with macrophages marker CD68 (red) or F4/80 (red), Tim-4 (green), and nuclei marker DAPI (blue) in IVCs of DVT mice. Scale bar, 200 μm. **(E)** FCM analysis showing the percentage of Tim-4 in F4/80^+^CD11b^+^Ly6C^+^Ly6G^low^ cells from spleen and F4/80^+^CD11b^+^ cells from PEMs of DVT mice on days 0, 3, 5, 7, 11, and 15. **(F)** qPCR analysis for Tim-4, IL-1β, and TNF-α in THP-1 with the stimulation of conditioned medium from DVT patients or control subjects, respectively. **(G)** Western blot analysis of Tim-4, pP65, and P65 expression in THP-1 and HUVECs co-culture system with incubation of conditioned medium from DVT patients or control subjects, respectively. Error bars indicate SD of at least three biological replicates per group in one experiment. *p < 0.05, **p < 0.01, ***p < 0.001, ****p < 0.0001.

To identify the cellular source of differentially expressed Tim-4 in IVCs from Sham and DVT mice, single-cell transcriptome sequencing analysis on IVCs from mice with DVT or Sham operation retrieved from the GEO database (GSE221978) was performed. 9 distinct cell populations composed of multiple subpopulations were identified and annotated in **Figure 1A**, and cell distributions in sham and DVT groups were displayed respectively in **Supplementary Figure S3A**. Subsequently, t-distributed stochastic neighbor embedding (t-SNE) analysis showed the relative distribution of Tim-4 in visual map of different cell clusters. Impressively, we found that Tim-4 was the highest enriched in macrophages among all individual vein wall cell types at basal expression levels, and Tim-4 was decreased in macrophages from DVT mice compared with Sham group (**Figure 1B**; **Supplementary Figure S3B**). Therefore, we further explored the expression and function of Tim-4 in macrophages from IVCs. Consistent with above results, IF staining results revealed that Tim-4 expression in CD68^+^ or F4/80^+^ macrophages exhibited an initial downregulation followed by an increasing tendency over time (**Figures 1C, D**). To unravel the involvement of Tim-4 in immunity, we further detected expression of Tim-4 in macrophages from spleens, and peritoneal fluid of DVT and Sham mice by FCM. As shown in **Figure 1E**, Tim-4 expression also displayed a similar trend of decreasing first and then increasing in F4/80^+^CD11b^+^Ly6C^+^Ly6G^low^ macrophages from spleens, and F4/80^+^CD11b^+^ PEMs of DVT mice. More interestingly, Tim-4 expression level was decreased dramatically since third day after modeling, which drove us to select the third day as observation time point to explore the underlying mechanisms in the following studies. Similar results were obtained *in vitro* from a THP-1 and HUVECs co-culture system stimulated with serum from DVT patients that Tim-4 was downregulated, whereas IL-1β and TNF-α were upregulated compared with controls in THP-1 cells (**Figure 1F**). Meanwhile, pP65 was increased in THP-1 treated with DVT derived-conditioned medium (**Figure 1G**). Collectively, above findings demonstrated that Tim-4 expression in macrophages was downregulated during the early stage of DVT, whereas proinflammatory factors were upregulated, implying that macrophage Tim-4 might play a critical role in DVT pathogenesis by modulating the secretion of inflammatory factors through NF-κB activation.

### 3.2 Tim-4 deficiency in macrophages augments thrombosis *in vitro* and *in vivo*

**Figure 2:**
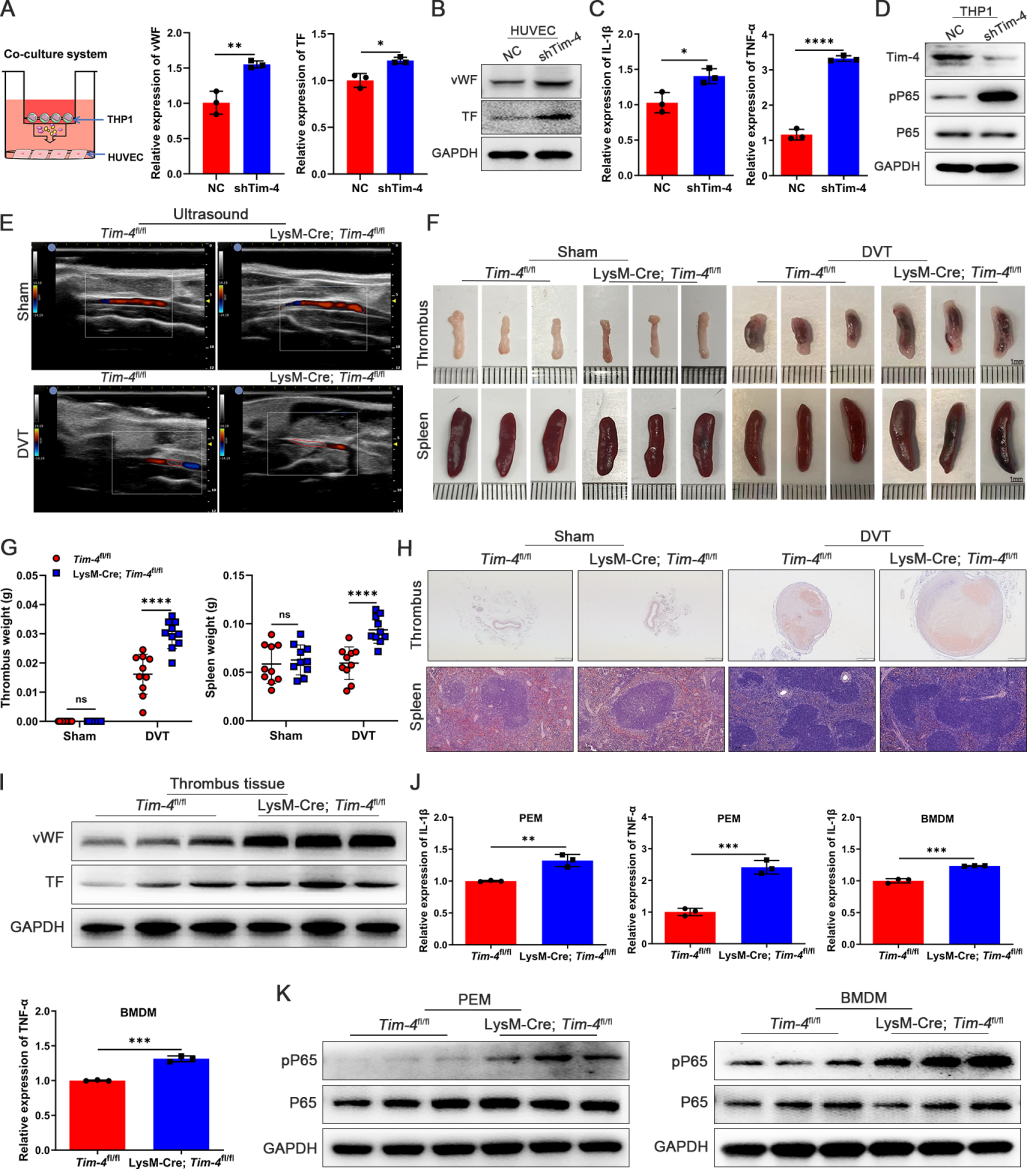
Tim-4 deficiency in macrophages leads to enhanced thrombosis *in vitro* and *in vivo*. **(A-D)** THP-1 cells were transfected with Lv-shTim-4 or Lv-NC for 72 hours, treated with LPS for another 3.5 hours, and co-cultured with HUVECs. **(A)** Model diagram of THP-1 and HUVECs co-culture system and relative mRNA levels of vWF and TF in HUVECs. **(B)** Western blot analysis of vWF and TF in HUVECs. **(C)** Relative mRNA levels of IL-1β and TNF-α in THP-1. **(D)** Western blot analysis of Tim-4, pP65, and P65 expression in THP-1. **(E-H)** Representative ultrasound images, photographs (Scale bar, 1 mm) and weight statistics of thrombi and spleens, as well as HE-stained sections of thrombi (scale bar, 500 μm) and spleens (scale bar, 0.100 mm), were obtained from sham and DVT group mice on day 3. **(I)** Western blot analysis of vWF and TF expression in IVCs from *Tim-4* ^fl/fl^ and LysM-Cre; *Tim-4* ^fl/fl^ mice, respectively. **(J)** Relative mRNA levels of IL-1β and TNF-α in PEMs and BMDMs from *Tim-4* ^fl/fl^ and LysM-Cre; *Tim-4* ^fl/fl^ mice. **(K)** Western blot analysis of pP65 and P65 expression in PEMs and BMDMs from *Tim-4* ^fl/fl^ and LysM-Cre; *Tim-4* ^fl/fl^ mice, respectively. Error bars indicate SD of at least three biological replicates per group in one experiment. *p < 0.05, **p < 0.01, ***p < 0.001, ****p < 0.0001, ns, no significance.

To investigate the potential function of macrophage Tim-4 in DVT progression, we first successfully silenced the expression of Tim-4 in THP-1 *in vitro*, and Tim-4 could be effectively downregulated ~80% when transfected with Lv-shTim-4 compared with Lv-NC (**Supplementary Figure S4A**). In the THP-1 and HUVECs co-culture system, the silencing of Tim-4 significantly augmented the expression of vWF, TF, and ICAM-1 in HUVECs (**Figures 2A, B**; **Supplementary Figure S4B**), accompanied with increased pro-inflammatory factors (IL-1β and TNF-α) and the phosphorylation of P65 in co-cultured THP-1 cells for responding to Tim-4 inhibition (**Figures 2C, D**). Next, to further investigate the role of Tim-4 in macrophages *in vivo*, we successfully constructed macrophage-specific *Tim-4* knockout mice (LysM-Cre; *Tim-4* ^fl/fl^). Successful knockout was confirmed by genotyping (**Supplementary Figure S4C**) and analysis of Tim-4 expression in primary macrophages of PEMs and BMDMs by PCR, qPCR, western blot, and FCM analyses (**Supplementary Figures S4D-G**). Then DVT models were established in *Tim-4* ^fl/fl^ and LysM-Cre; *Tim-4* ^fl/fl^ mice respectively. In the DVT group, macrophage conditional *Tim-4* knockout mice exhibited enlarged and increased weights of thrombi and spleens, increased cross-sectional area and more inflammatory foci in thrombi, and more red blood cells infiltrating the red pulp area of the spleens demonstrated by color doppler ultrasounds, photographs, and HE staining results compared with *Tim-4* ^fl/fl^ mice. However, there was no significant difference between *Tim-4* ^fl/fl^ and LysM-Cre; *Tim-4* ^fl/fl^ mice in the sham group (**Figures 2E-H**). However, the expression of vWF and TF in IVCs of LysM-Cre; *Tim-4* ^fl/fl^ mice was significantly elevated compared with *Tim-4* ^fl/fl^ mice by western blot and IHC assays (**Figure 2I**; **Supplementary Figure S5A**). Consistently, the expression of pro-inflammatory factors (IL-1β and TNF-α) in PEMs and BMDMs derived from LysM-Cre; *Tim-4* ^fl/fl^ mice was significantly augmented verified by qPCR and ELISA assays, respectively (**Figure 2J**; **Supplementary Figure S5B**), which were accompanied with elevated phosphorylation level of P65 (**Figure 2K**). These results suggested that the macrophage-specific *Tim-4* deficiency exacerbated inflammatory responses in macrophages and enhanced prothrombogenic factors expression in endothelial cells, which might be related to triggering NF-κB pathway activation and ultimately facilitate thrombus formation.

### 3.3 Macrophage Tim-4 mitigates inflammation-induced thrombosis in DVT through NF-κB pathway

**Figure 3:**
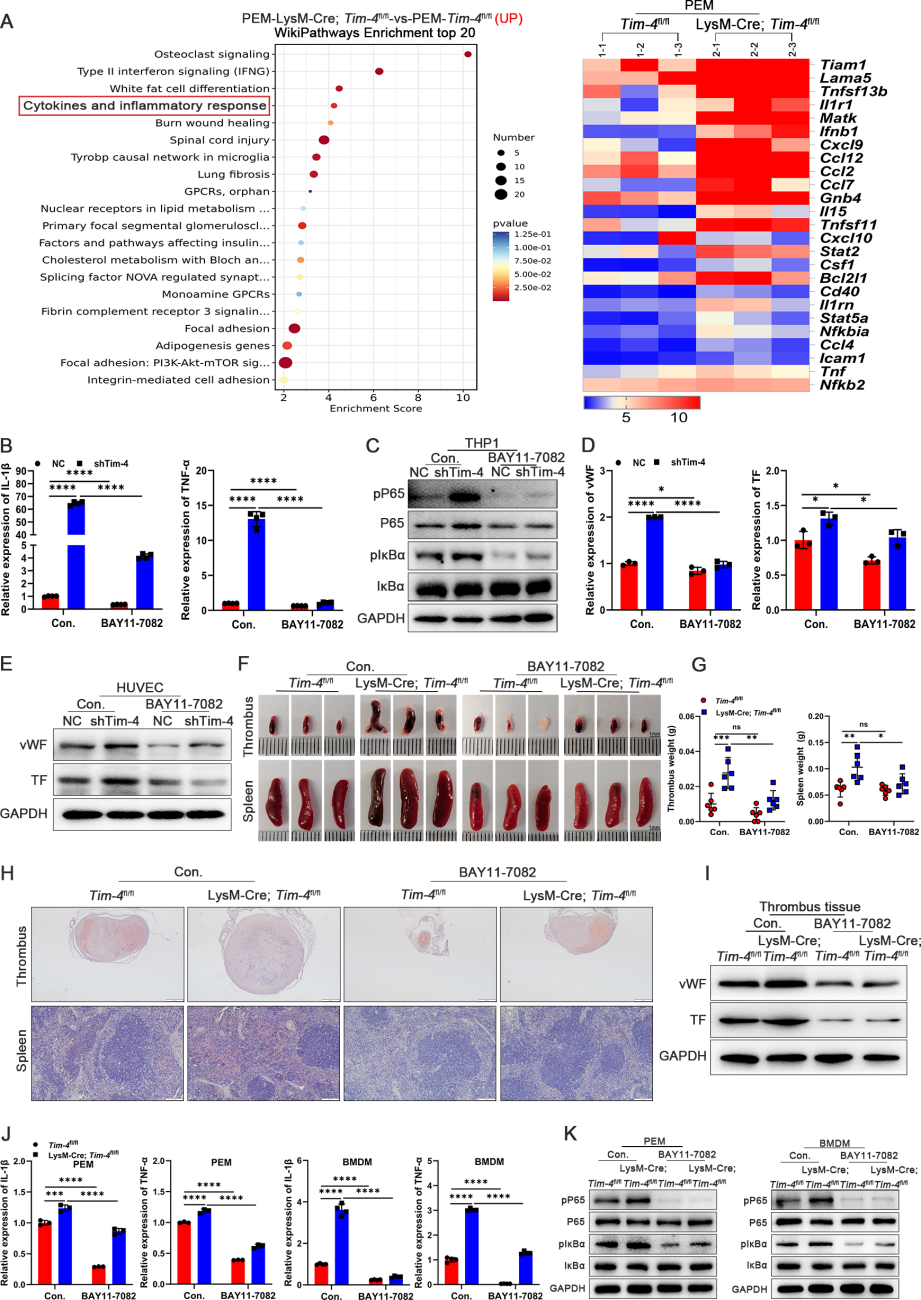
Macrophage Tim-4 inhibits DVT formation through the NF-κB signaling pathway. **(A)** The top 20 Wikipathway-enriched pathways (up) and heat map of genes related cytokines and inflammatory response from PEMs in RNA sequencing. **(B-E)** THP-1 cells were transfected with Lv-shTim-4 or Lv-NC for 72 hours and treated with LPS for 0.5 hours, and inhibitor groups were treated with BAY11-7082 for another 1 hour and co-cultured with HUVECs. **(B)** Relative mRNA levels of IL-1β and TNF-α in THP-1. **(C)** Western blot analysis of pP65, P65, pIκBα, and IκBα expression in THP-1. **(D)** Relative mRNA levels of vWF and TF in HUVECs co-cultured with THP-1. **(E)** Western blot analysis of vWF and TF in HUVECs co-cultured with THP-1. **(F-H)** Representative images (Scale bar, 1 mm), measurement results of weight, and representative HE staining of thrombi (scale bar, 500 μm) and spleens (scale bar, 200 μm) from *Tim-4* ^fl/fl^ and LysM-Cre; *Tim-4* ^fl/fl^ mice with or without BAY11-7082 treatment. **(I)** Western blot analysis of vWF and TF expression in thrombus tissues from *Tim-4* ^fl/fl^ and LysM-Cre; *Tim-4* ^fl/fl^ mice with or without BAY11-7082 treatment, respectively. **(J)** qPCR analysis for IL-1β and TNF-α in PEMs and BMDMs from *Tim-4* ^fl/fl^ and LysM-Cre; *Tim-4* ^fl/fl^ mice with or without BAY11-7082 treatment, respectively. **(K)** Western blot analysis of pP65, P65, pIκBα, and IκBα expression in PEMs and BMDMs from *Tim-4* ^fl/fl^ and LysM-Cre; *Tim-4* ^fl/fl^ mice with or without BAY11-7082 treatment, respectively. Error bars indicate SD of at least three biological replicates per group in one experiment. *p < 0.05, **p < 0.01, ***p < 0.001, ****p < 0.0001, ns, no significance.

To elucidate the potential pathway by which *Tim-4* deficiency in macrophages accelerated DVT pathogenesis, we isolated primary PEMs from *Tim-4* ^fl/fl^ and LysM-Cre; *Tim-4* ^fl/fl^ mice for RNA sequencing. Interestingly, top 20 WikiPathways Enrichment analyses identified a significant enrichment of the cytokine-mediated inflammatory response pathway (ranked 4th), which was particularly relevant to DVT pathogenesis. Corresponding heatmap analysis demonstrated upregulation of pathway-associated differentially expressed genes (**Figure 3A**, GSE274504). Moreover, *Tim-4* knockout in macrophages further promoted phosphorylation of P65, which also prompted us to verify whether Tim-4 might regulate the inflammatory response through the NF-κB pathway. To clarify the potential role of NF-κB signaling in the Tim-4-mediated macrophage inflammatory response in DVT, an NF-κB inhibitor (BAY11-7082) was introduced to treat macrophages under stimulation with LPS *in vitro*. *Tim-4* knockdown in co-cultured THP-1 (**Supplementary Figures S6A, B**) increased the expression of pro-inflammatory factors (IL-1β and TNF-α) and phosphorylation of P65 (Ser529) and IκBα (Ser32) in THP-1 cells, as well as prothrombotic factors (vWF, TF, and ICAM-1) in HUVECs, whereas these manners of *Tim-4* knockdown-dependent pro-thrombosis disappeared after BAY11-7082 administration (**Figures 3B-E**; **Supplementary Figure S6C**). Above findings prompted us to elucidate the role of Tim-4 in regulating the NF-κB signaling pathway *in vivo*, and then BAY11-7082 was intraperitoneally injected into LysM-Cre; *Tim-4* ^fl/fl^ mice or *Tim-4* ^fl/fl^ mice respectively. Consistent with the *in vitro* experiments, we found that LysM-Cre; *Tim-4* ^fl/fl^ mice promoted inflammation, and thrombosis development. However, these effects were abolished following treatment with BAY11-7082. More specifically, BAY11-7082 treatment profoundly alleviated thrombosis in both LysM-Cre; *Tim-4* ^fl/fl^ mice or *Tim-4* ^fl/fl^ mice demonstrated by photographs, HE staining, western blot, and IHC analyses (**Figures 3F-I**; **Supplementary Figure S6D**). The anti-thrombotic effects correlated with attenuated expression of pro-inflammatory factors (IL-1β and TNF-α), and reduced phosphorylation of P65 and IκBα in both PEMs and BMDMs compared to untreated controls (**Figures 3J, K**; **Supplementary Figure S6E**), consistent with inflammation-driven thrombosis. Above results indicated that *Tim-4* deletion in macrophages exacerbated DVT progression by augmenting NF-κB signaling-mediated inflammatory responses, as evidenced by reversible effects after NF-κB inhibitor treatment.

### 3.4 Tim-4 suppresses NF-κB pathway activation by hijacking CK2β

**Figure 4:**
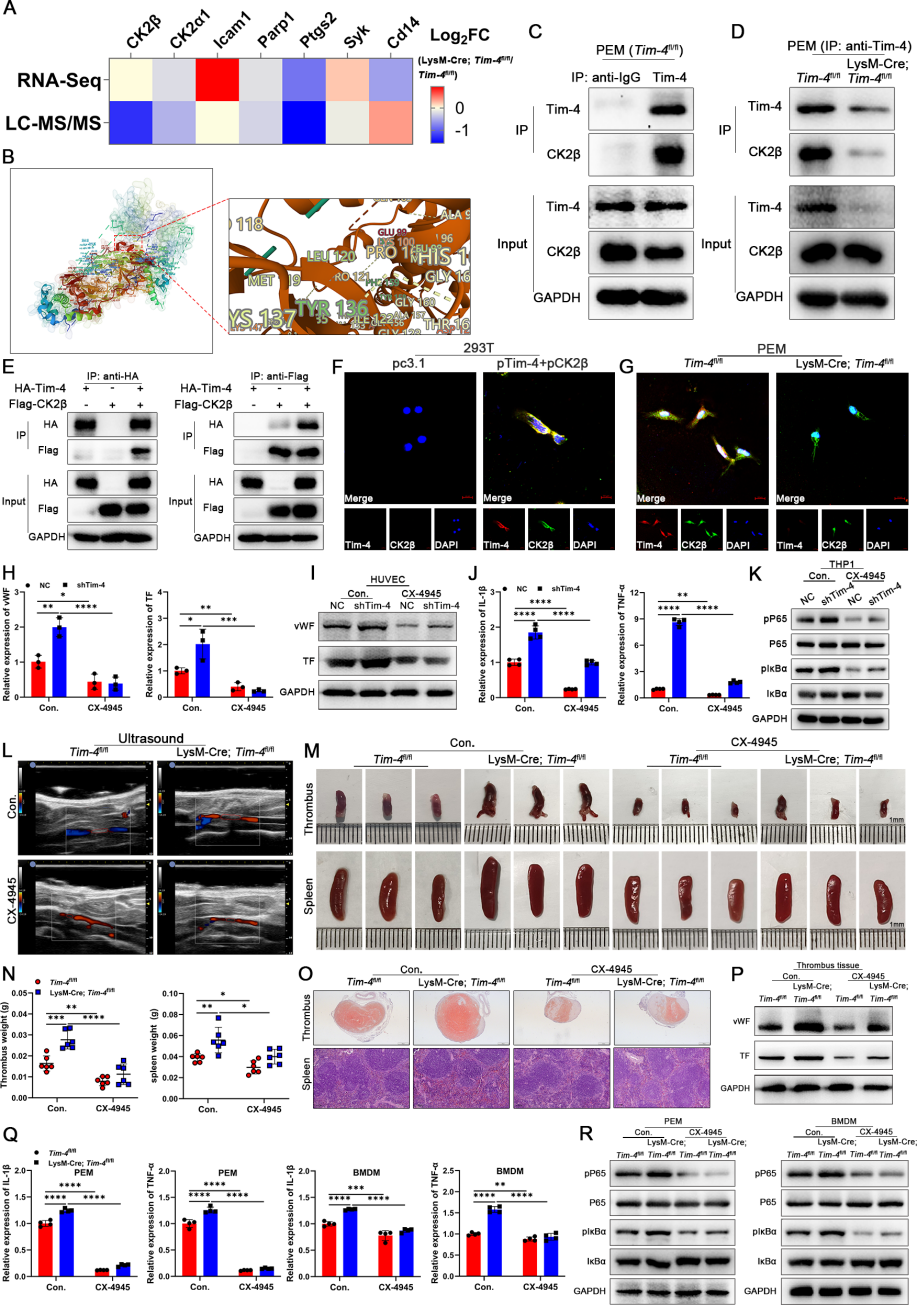
Tim-4 hijacks CK2β to prevent the activation of the NF-κB pathway. **(A)** Tim-4 binding proteins in PEMs were identified by RNA-Seq combined with LC-MS/MS assay. **(B)** Predicted docking module of Tim-4 and CK2β. **(C, D)** IP validation of Tim-4 binding to CK2β in PEMs. **(E)** Co-IP assay using either anti-HA or anti-Flag antibodies with lysates from HEK293T cells 48 hours after co-transfection with HA-tagged Tim-4 and Flag-tagged CK2β plasmids. **(F, G)** Representative colocalization IF images of Tim-4 and CK2β in HEK293T cells or PEMs. Scale bar, 10μm. **(H-K)** THP-1 cells were transfected with Lv-shTim-4 or Lv-NC for 72 hours and treated with LPS for 0.5 hours, and inhibitor groups were treated with CX-4945 for another 1 hour and co-cultured with HUVECs. **(H, I)** Relative mRNA and protein levels of vWF and TF in HUVECs. **(J, K)** Relative mRNA levels of IL-1β and TNF-α, and western blot analysis of pP65, P65, pIκBα, and IκBα expression in THP-1. **(L-O)** Representative ultrasound images and photographs (Scale bar, 1 mm) of thrombi, weight statistics, and HE staining of thrombi (scale bar, 500 μm) and spleens (scale bar, 0.100 mm) from *Tim-4* ^fl/fl^ and LysM-Cre; *Tim-4* ^fl/fl^ mice with or without CX-4945 treatment. **(P)** Western blot analysis of vWF and TF expression in IVCs from *Tim-4* ^fl/fl^ and LysM-Cre; *Tim-4* ^fl/fl^ mice with or without CX-4945 treatment. **(Q)** qPCR analysis for IL-1β and TNF-α in PEMs and BMDMs from *Tim-4* ^fl/fl^ and LysM-Cre; *Tim-4* ^fl/fl^ mice with or without CX-4945 treatment. **(R)** Western blot analysis of pP65, P65, pIκBα, and IκBα expression in PEMs and BMDMs from *Tim-4* ^fl/fl^ and LysM-Cre; *Tim-4* ^fl/fl^ mice with or without CX-4945 treatment. Error bars indicate SD of at least three biological replicates per group in one experiment. *p < 0.05, **p < 0.01, ***p < 0.001, ****p < 0.0001.

To further elucidate the molecular mechanism underlying the inhibitory effects of Tim-4 on activation of NF-κB pathway, Co-IP assay coupled with LC-MS/MS was performed to identify Tim-4 binding proteins in PEMs. LC-MS/MS assay identified CK2β (a regulatory subunit of CK2) as the most significantly altered Tim-4-interacting protein, despite unaltered transcriptional expression in RNA-Seq assay from PEMs, strongly indicating its role as a critical Tim-4-binding partner at the protein level (**Figure 4A**, IPX0012568000). ZDOCK-based rigid protein–protein docking was performed between proteins Tim-4 and CK2β, and predicted a stable interaction, supported by favorable Z-scores and optimal binding conformation presented in **Supplementary Figure S7A**; **Figure 4B**, suggesting a formation of direct molecular association. Besides, comparative sequence alignment analysis showed complete conservation (100% identity) of CK2β between human and mouse orthologs at the amino acid level (**Supplementary Figure S7B**). Co-IP assay confirmed the interaction between endogenous Tim-4 and CK2β in PEMs (**Figures 4C, D**) and between ectopically expressed HA-tagged Tim-4 and Flag-tagged CK2β in HEK293T cells (**Figure 4E**). Furthermore, confocal assay demonstrated significant colocalization of Tim-4 and CK2β in both PEMs and HEK293T cells (**Figures 4F, G**). Taken together, these results established Tim-4 as a direct binding partner of CK2β.

To investigate whether Tim-4 suppressed NF-κB pathway activation by sequestering CK2β, CX-4945 (a selective CK2 inhibitor under clinical trials for cancer therapy) was employed both *in vitro* and *in vivo*. The results from co-culture system of THP-1 and HUVECs *in vitro* showed that *Tim-4* knockdown in THP-1 significantly enhanced a prothrombotic phenotype in HUVECs (increased vWF and TF expression at mRNA and protein levels) while augmenting NF-κB activation in macrophages (elevated expression of IL-1β, TNF-α, pP65 and pIκBα) in THP-1, whereas these effects were largely abrogated after treatment with CX-4945 (**Figures 4H-K**). Consistent with *in vitro* findings, macrophage-specific *Tim-4* knockout mice exhibited exacerbated DVT progression *in vivo*, accompanied by enhanced NF-κB activation (increased phosphorylation of P65 and IκBα) and elevated pro-inflammatory factors (IL-1β and TNF-α) in both PEMs and BMDMs. These effects were effectively reversed by CX-4945 treatment both in LysM-Cre; *Tim-4* ^fl/fl^ mice and *Tim-4* ^fl/fl^ mice (**Figures 4L-R**). Collectively, these data demonstrated that Tim-4, as a natural CK2 inhibitor, hijacked CK2β from the holoenzyme, thereby efficiently diminishing CK2-mediated phosphorylation of P65 and IκBα, and ultimately restraining thrombogenesis.

### 3.5 miR-93-5p directly targets and suppresses Tim-4 expression

**Figure 5:**
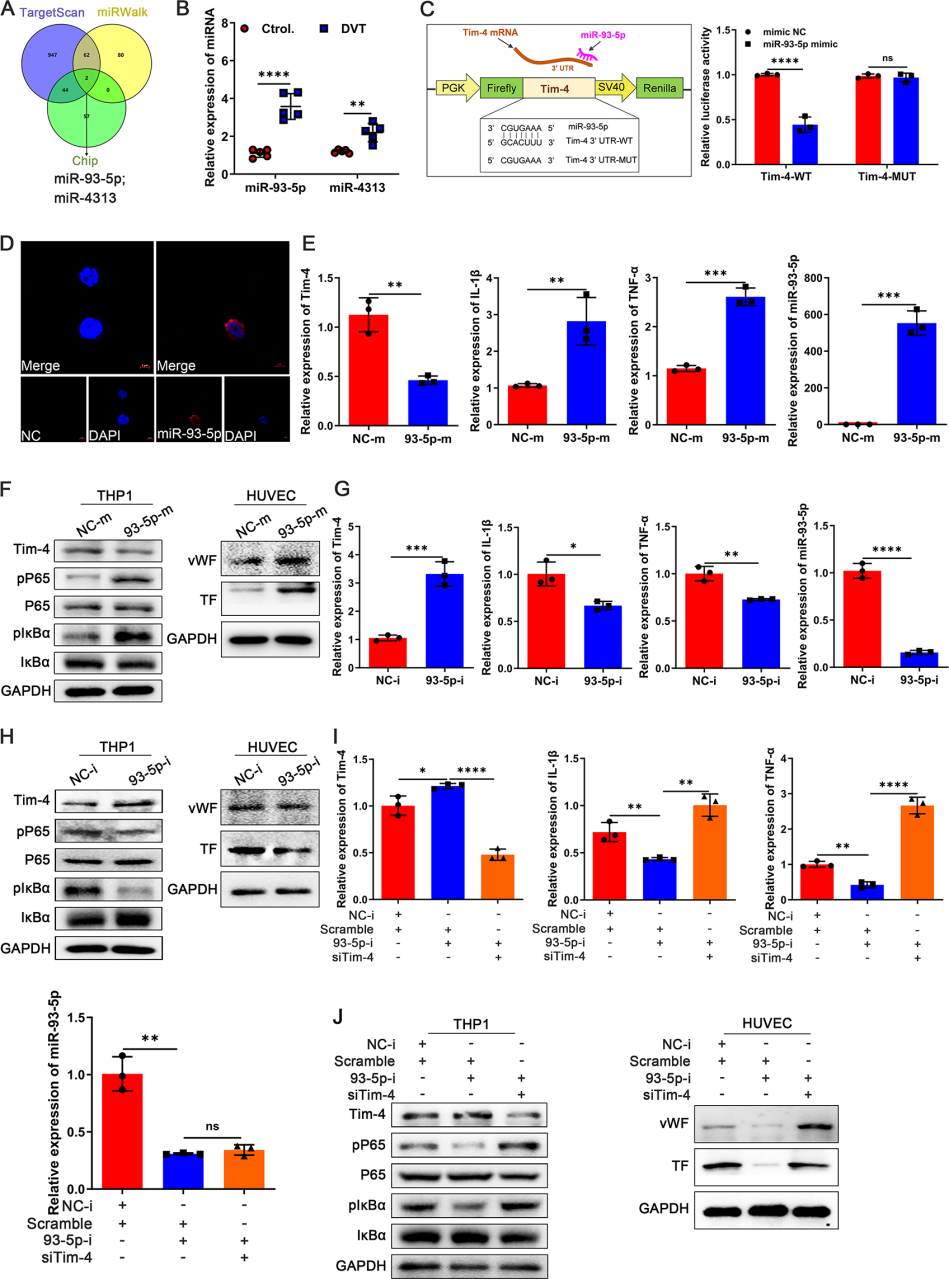
Identification of Tim-4 as a direct target of miR-93-5p. **(A)** TargetScan, miRWalk, and Chip databases were used to predict Tim-4-targeted miRNAs. **(B)** qPCR analysis for miR-93-5p and miR-4313 in PBMCs from DVT patients or control subjects, respectively. **(C)** Tim-4 3′UTR predicted regions targeted by miR-93-5p and the luciferase reporter assay of the relationship between miR-93-5p mimic and 3′UTR of Tim-4. **(D)** RNA FISH of miR-93-5p (red) in THP-1. Nuclei were stained with DAPI (blue). Scale bar: 5 μm. **(E)** qPCR analysis for Tim-4, IL-1β, TNF-α, and miR-93-5p in THP-1 transfected with miR-93-5p mimic or negative control mimic for 48 hours, respectively. **(F)** Western blot analysis of Tim-4, pP65, P65, pIκBα, and IκBα expression in THP-1 transfected with miR-93-5p mimic or negative control mimic for 48 hours and western blot analysis of vWF and TF in HUVECs co-cultured with THP-1, respectively. **(G)** qPCR analysis for Tim-4, IL-1β, TNF-α, and miR-93-5p in THP-1 transfected with miR-93-5p inhibitor or negative control inhibitor for 48 hours, respectively. **(H)** Western blot analysis of Tim-4, pP65, P65, pIκBα, and IκBα expression in THP-1 transfected with miR-93-5p inhibitor or negative control inhibitor for 48 hours and western blot analysis of vWF and TF in HUVECs co-cultured with THP-1. **(I, J)** THP-1 cells were transfected with siTim-4 or scramble for 48 hours and then transduced with miR-93-5p inhibitor or negative control inhibitor for 48 hours, followed by co-cultivation with HUVECs. **(I)** qPCR analysis for Tim-4, IL-1β, TNF-α, and miR-93-5p in THP-1 described as above. **(J)** Western blot analysis of Tim-4, pP65, P65, pIκBα, and IκBα expression in THP-1 and western blot analysis of vWF and TF in HUVECs co-cultured with THP-1 as described above. Error bars indicate SD of at least three biological replicates per group in one experiment. NC-m, negative control mimic, 93-5p-m, miR-93-5p mimic, NC-i, negative control inhibitor, 93-5p-i, miR-93-5p inhibitor. *p < 0.05, **p < 0.01, ***p < 0.001, ****p < 0.0001, ns, no significance.

Studies have demonstrated that miRNAs play a critical role in post-transcriptional gene silencing and are involved in modulating various inflammatory and immune responses (41). Given this, we next investigated whether Tim-4 downregulation during DVT progression was mediated by upregulated miRNAs. To identify potential miRNAs targeting Tim-4 3’UTR, we performed a comprehensive bioinformatics analysis using TargetScan, miRWalk, and Chip databases. Based on this screening, two candidate miRNAs of Tim-4, miR-93-5p and miR-4313, were predicted to bind Tim-4 and selected for further validation (**Figure 5A**). Given the profound differential expression of miR-93-5p in PBMCs from DVT patients compared with healthy controls (**Figure 5B**), and the absence of a murine ortholog for miR-4313 in miRDB, miRWalk, and TargetScan databases until now, we focused subsequent investigations on miR-93-5p. Bioinformatics analysis using miRanda and TargetScan predicted a conserved binding site for miR-93-5p within the wild-type Tim-4 3′UTR (WT), and Tim-4 3′UTR MUT represents the UTR in which the binding site for miR-93-5p was mismatched mutation. Luciferase reporter assay showed that miR-93-5p mimics significantly suppressed luciferase activity in HEK293T cells transfected with the wild-type Tim-4 3′UTR, whereas this inhibitory effect was abolished upon introduction of the Tim-4 3′UTR MUT (**Figure 5C**). Moreover, RNA FISH assay confirmed predominant cytoplasmic localization of miR-93-5p compared with control tested by confocal microscopy (**Figure 5D**). To assess the direct regulatory role of miR-93-5p on Tim-4, we subsequently transfected THP-1 cells with either miR-93-5p mimic or inhibitor in the THP-1 and HUVECs co-culture system. The results showed that overexpression of miR-93-5p significantly suppressed Tim-4 expression, while concurrently upregulating proinflammatory mediators (IL-1β, TNF-α), phosphorylation of NF-κB pathway components (P65, IκBα), and miR-93-5p levels in THP-1 cells. Notably, miR-93-5p overexpression in THP-1 cells also enhanced the expression of vWF and TF in co-cultured HUVECs (**Figures 5E, F**). Conversely, miR-93-5p inhibitor up-regulated Tim-4 expression, which correlated with suppressed inflammatory factors (IL-1β, TNF-α) and NF-κB signaling (reduced P65 and IκBα phosphorylation) in THP-1 cells, along with attenuated vWF and TF production in co-cultured HUVECs (**Figures 5G, H**). To evaluate whether miR-93-5p regulated inflammatory responses and thrombosis through Tim-4, we performed rescue experiments by co-transfecting miR-93-5p inhibitor and lentivirus-siTim-4 into THP-1 cells. As expected, miR-93-5p inhibition upregulated Tim-4 while reducing levels of inflammatory cytokines (IL-1β, TNF-α) and phosphorylation of P65 and IκBα in THP-1 cells, along with decreased expression of vWF and TF in co-cultured HUVECs. Importantly, these anti-inflammatory and antithrombotic effects were reversed by concomitant Tim-4 knockdown, demonstrating that Tim-4 was the essential downstream effector of miR-93-5p-mediated inflammatory activation and thrombus formation (**Figures 5I, J**). Taken together, these data identified miR-93-5p as a novel negative regulator of Tim-4 in macrophage inflammatory responses.

### 3.6 LncRNF219-3:1 upregulates Tim-4 indirectly by sponging miR-93-5p

**Figure 6:**
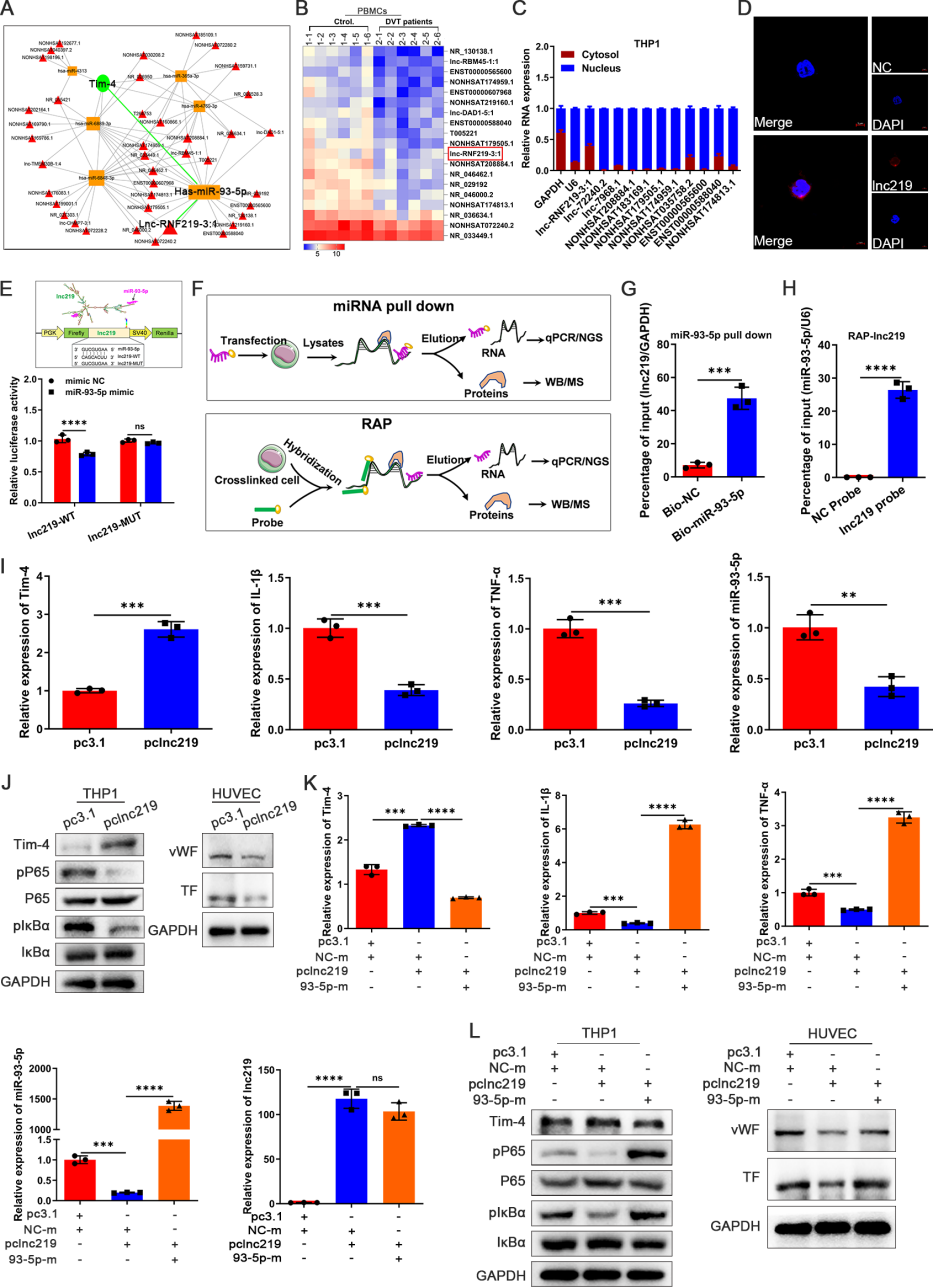
LncRNF219-3:1 sponges miR-93-5p to increase Tim-4 levels and reduce macrophage inflammation. **(A)** The lncRNA-miRNA-mRNA regulation network. **(B)** Heat map showing differential expression of lncRNAs between PBMCs isolated from DVT patients and control subjects related to lncRNA-miRNA-mRNA regulation network (n = 6). **(C)** Expression levels of lncRNAs in the cytosol and nucleus respectively in THP-1. **(D)** RNA FISH of lncRNF219-3:1 (lnc219, red) in THP-1. Nuclei were stained with DAPI (blue). Scale bar: 5 μm. **(E)** Lnc219 3′UTR predicted regions targeted by miR-93-5p and luciferase reporter assay of the relationship between miR-93-5p mimic and 3′UTR of lnc219. **(F)** Patterns of miRNA pull down and RAP. **(G)** THP-1 cells were transfected with Bio-NC and Bio-miR-93-5p-WT for 48 hours, followed by pull-down assay. **(H)** After the RAP assay, relative expression level of miR-93-5p was detected by qPCR. **(I)** qPCR analysis for Tim-4, IL-1β, TNF-α, and miR-93-5p in THP-1 transfected with pc3.1 or pclnc219 for 48 hours, respectively. **(J)** Western blot analysis of Tim-4, pP65, P65, pIκBα, and IκBα expression in THP-1 transfected with pc3.1 or pclnc219 for 48 hours and western blot analysis of vWF and TF in HUVECs co-cultured with THP-1, respectively. **(K, L)** THP-1 cells were transfected with pclnc219 or pc3.1 for 48 hours and then transduced with miR-93-5p mimic or negative control mimic for 48 hours, followed by co-cultivation with HUVECs. **(K)** qPCR analysis for Tim-4, IL-1β, TNF-α, miR-93-5p, and lnc219 in THP-1 as described above. **(L)** Western blot analysis of Tim-4, pP65, P65, pIκBα, and IκBα expression in THP-1 and western blot analysis of vWF and TF in HUVECs co-cultured with THP-1 described as above. Error bars indicate SD of at least three biological replicates per group in one experiment. NC-m, negative control mimic, 93-5p-m, miR-93-5p mimic. **p < 0.01, ***p < 0.001, ****p < 0.0001, ns, no significance.

Considering lncRNAs can regulate gene expression by functioning as ceRNAs that competitively binding to miRNAs (42), we further investigated whether the elevated miR-93-5p levels observed in DVT might be modulated by potential lncRNAs, consequently suppressing Tim-4 expression and thrombotic progression. We first systematically constructed a lncRNA-miRNA-mRNA regulatory network (involving 40 lncRNAs and 6 miRNAs) by an integrated bioinformatics approach to identify potential regulators of the miR-93-5p/Tim-4 axis (**Figure 6A**), and 19 putative lncRNAs were selected that were downregulated in microarray analysis (**Figure 6B**). Among them, we excluded 8 putative lncRNAs due to unsuitable primer sequences for subsequent analysis, and successfully developed primer pairs to assess their subcellular localization in the nucleus and cytoplasm for remaining 11 lncRNAs. Integrated analysis revealed that lncRNF219-3:1 (NONHSAT166973.1, chr13: 79890530-79890471) exhibited predominant cytoplasmic localization in THP-1 cells, as confirmed by qPCR analysis of nuclear and cytoplasmic fractions, RNA FISH and lncLocator prediction database (**Figures 6C, D**; **Supplementary Figure S8A**). Moreover, lncRNF219-3:1 also showed minimal protein-coding potential (Coding probability~0.0015934) and absence of coding label analyzed by a coding potential assessment tool (**Supplementary Figure S8B**), which strongly suggested that lncRNF219-3:1 was a lead candidate. Then, a conserved miR-93-5p binding site in lncRNF219-3:1 was predicted and analyzed with miRanda and TargetScan databases. Luciferase reporter assay indicated that miR-93-5p mimic significantly suppressedthat lncRNF219-3:1-WT reporter activity, whereas this effect was completely abolished within lncRNF219-3:1-MUT, confirming a direct binding site (**Figure 6E**). To further validate the direct interaction between miR-93-5p and lncRNF219-3:1 in THP-1 cells, we performed complementary RNA pull-down assays. The miRNA pull-down assay showed that biotinylated miR-93-5p (Bio-miR-93-5p) could significantly enrich more lncRNF219-3:1 compared to the control probe. Consistently, the RNA antisense purification (RAP) assay using a biotinylated lncRNF219-3:1 probe demonstrated a remarkable miR-93-5p enrichment, indicating a direct and physical association between lncRNF219-3:1 and miR-93-5p (**Figures 6F-H**). To further explore the biological function of identified lncRNF219-3:1, a full-length recombinant plasmid (pclncRNF219-3:1) and small interfering RNA (silncRNF219-3:1) were used to specifically increase and knockdown lncRNF219-3:1 expression respectively (**Supplementary Figures S8C, D**). LncRNF219-3:1 overexpression significantly increased Tim-4 expression while decreasing inflammatory factors (IL-1β, TNF-α) and phosphorylation of P65/IκBα in THP-1 cells, with concordant reductions of prothrombotic markers (vWF and TF) in co-cultured HUVECs (**Figures 6I, J**). Conversely, lncRNF219-3:1 knockdown elicited opposing effects in both THP-1 and co-cultured HUVECs (**Supplementary Figures S8E, F**). To establish miR-93-5p as the key mediator of lncRNF219-3:1’s effects, we performed reciprocal rescue experiments in the cocultrue system. Co-transfection with pclncRNF219-3:1 and miR-93-5p mimic completely abrogated lncRNF219-3:1-induced Tim-4 upregulation and reversed its suppressive effects on IL-1β, TNF-α, pP65, and pIκBα in THP-1 cells, and prothrombotic markers (vWF, TF) in co-cultured HUVECs (**Figures 6K, L**). Conversely, lncRNF219-3:1 knockdown phenotypes were reversed by miR-93-5p inhibition, establishing a linear lncRNF219-3:1/miR-93-5p/Tim-4 regulatory cascade (**Supplementary Figures S8G, H**). Collectively, these findings demonstrated that lncRNF219-3:1 functioned as a molecular sponge for miR-93-5p, thereby suppressing Tim-4 expression and inflammation in macrophages, ultimately attenuating thrombotic responses.

### 3.7 Clinical validation of Tim-4 and associated pathway targets expression profiling in DVT patient cohorts

**Figure 7:**
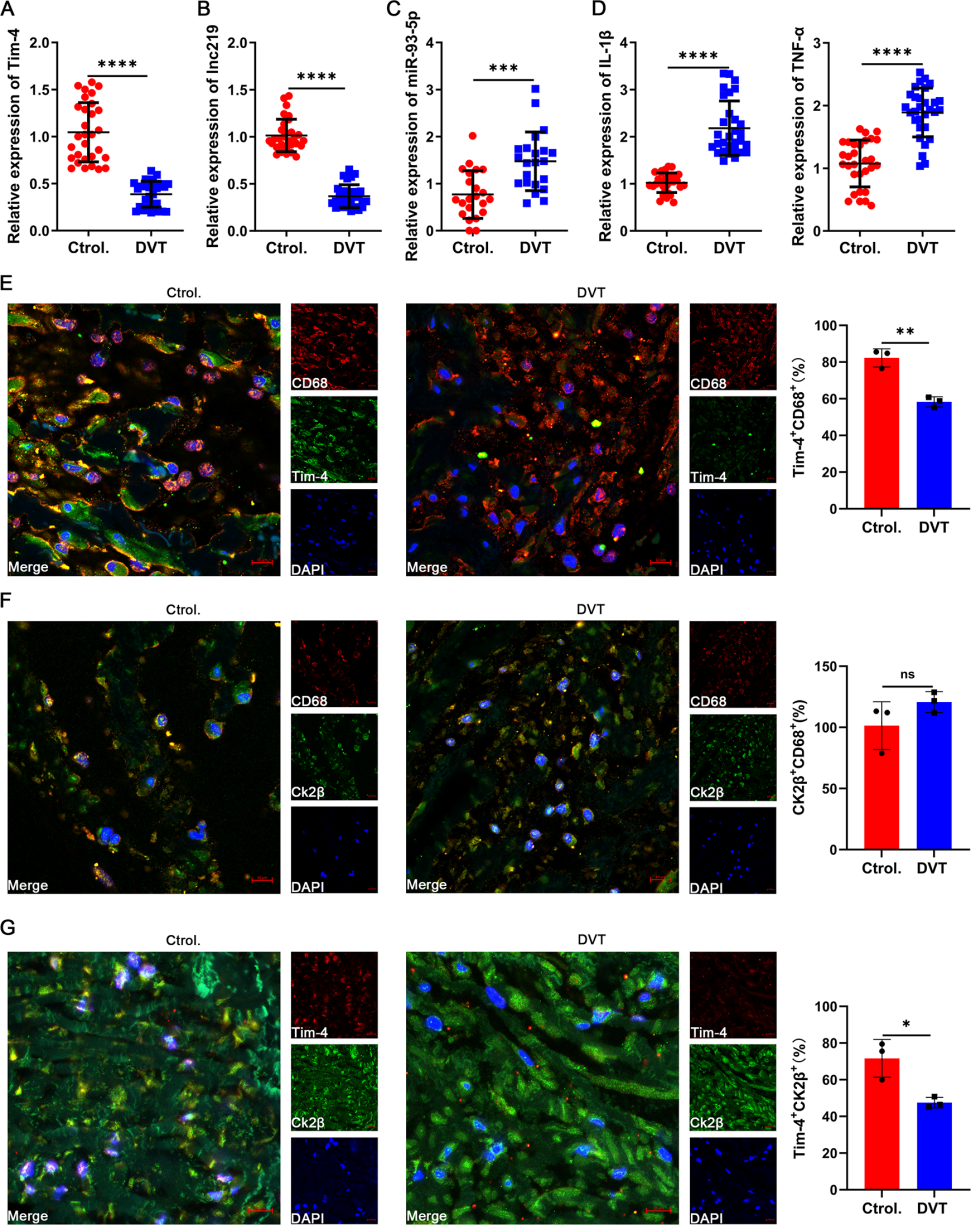
Confirmation of Tim-4 and associated molecule expression in clinical specimens. **(A-D)** qPCR assay for Tim-4, lnc219, miR-93-5p, IL-1β and TNF-α expression in PBMCs from DVT patients compared with control subjects at mRNA level. **(E-G)** IF staining and quantification of Tim-4 (green) and CD68 (red), CK2β (green) and CD68 (red), CK2β (green) and Tim-4 (red) respectively in popliteal vein tissues with or without thrombosis. Scale bar, 10 μm. Error bars indicate SD of at least three biological replicates per group in one experiment. *p < 0.05, **p < 0.01, ***p < 0.001, ****p < 0.0001, ns, no significance.

**Figure 8:**
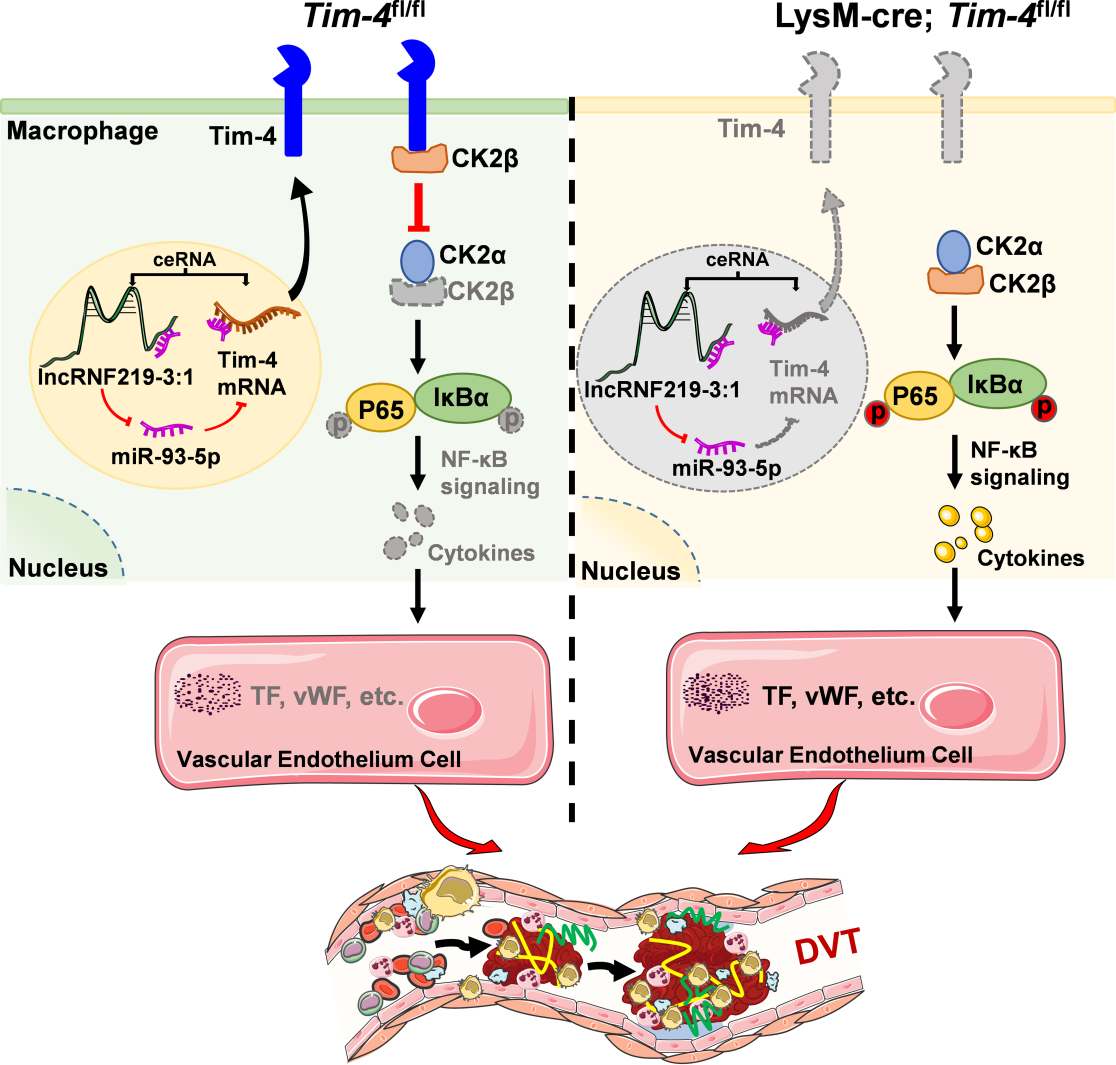
Schematic diagram of lncRNF219-3:1/miR-93-5p regulating Tim-4 in the pathogenesis of DVT by hijacking CK2β. In *Tim-4* ^fl/fl^ mice, highly expressed Tim-4 can inhibit kinase activity of CK2 holoenzyme by hijacking and interacting with CK2β, and diminish the phosphorylation of P65 and IκBα, thereby inhibiting NF-κB signaling pathway activation-induced DVT progression. In addition, highly expressed lnc219 acting as a competing endogenous RNA, can increase Tim-4 expression by sponging miR-93-5p and enhance Tim-4-mediated anti-inflammatory responses and anti-thrombosis effects. In lysM-Cre; *Tim-4* ^fl/fl^ mice, Tim-4 deficiency abolishes the interaction with CK2β, leading to activation of CK2 dependent NF-κB signaling pathway, and eventually aggravates inflammatory factors-mediated DVT development.

To clinically validate our above findings, we first isolated and analyzed PBMCs from peripheral blood of DVT patients and healthy controls. Notably, Tim-4 and lncRNF219-3:1 expression were significantly reduced in DVT specimens (~0.5 fold, **Figures 7A, B**), whereas miR-93-5p level and proinflammatory cytokines (IL-1β, TNF-α) were markedly elevated (~2 fold, **Figures 7C, D**) compared with healthy controls, consistent with our experimental results. To further validate our findings in human vascular tissue, we collected and analyzed a popliteal vein segment from an amputated DVT patient. The IF staining results revealed significantly reduced Tim-4 expression in CD68^+^ macrophages within IVCs compared to non-thrombosed vessels, while CK2β levels in CD68^+^ macrophages remained unchanged between groups, Tim-4/CK2β co-localization was markedly diminished in thrombosed segments (**Figures 7E-G**). These observations aligned with our *in vitro* and animal model data, confirming the clinical relevance of Tim-4 dysregulation in DVT pathogenesis. Together, our findings demonstrated that lncRNF219-3:1/miR-93-5p axis suppressed Tim-4 in macrophages, enhancing CK2β-dependent NF-κB activation through diminished Tim-4/CK2β interaction, thereby exacerbating DVT progression (**Figure 8**).

## 4 Discussion

Although macrophage-derived inflammatory factors have been established as key contributors to DVT pathogenesis (6), the precise regulatory mechanisms remain unclear. In the present study, we first characterize single-cell transcriptomic profiling of Tim-4 expression in inferior venous walls during acute DVT pathogenesis. Our analysis reveals a significant downregulation of Tim-4 in both tissue macrophages from murine DVT models and circulating PBMCs from DVT patients, suggesting its systemic involvement in thrombotic processes. Mechanistically, macrophage-specific *Tim-4* deletion exacerbates inflammatory responses by disrupting its physical interaction with CK2β, consequently activating NF-κB signaling and potentiating thrombus formation. Importantly, we identify a novel regulatory lncRNF219-3:1/miR-93-5p axis as a key upstream regulator of Tim-4 suppression in macrophages through a ceRNA-mediated mechanism. These findings substantially advance our understanding of DVT pathophysiology by: (1) establishing Tim-4 as a critical modulator and a potential therapeutic target for thrombotic disorders, and (2) elucidating a previously unrecognized Tim-4/CK2β interaction mechanisms and ncRNA-dependent regulatory circuit in DVT.

Macrophages are known to differentiate into two distinct phenotypes: the pro-inflammatory M1 type, and the anti-inflammatory, tissue-reparative M2 type (43). Recent studies indicate that pro-inflammatory macrophages dominate the early phase of DVT, which stabilize the thrombi through the release of inflammatory factors and promotion of fibrosis. In contrast, pro-resolving M2 macrophages facilitate thrombus resolution by enhancing fibrinolysis, collagen dissolution, and neovascularization during later stages (44). However, it should be noted that the temporal demarcation between early and late stages of DVT remains poorly defined. In our study, analysis up to day 15 post-DVT modeling revealed that thrombi maintained a homogeneous, non-fibrotic state during the first 7 days, followed by the onset of fibrosis and thrombolysis. Based on these observations, we define day 7 as a critical threshold: M1 predominated before day 7 (early phase) but showed fibrolytic activity thereafter. Consequently, we select day 3 post-modeling as the representative time point for subsequent studies. Notably, Tim-4 expression in thrombus tissue and macrophages exhibits a biphasic pattern, initially decreasing followed by rising progressively over time. These findings suggest that Tim-4 may play stage-specific and potentially dual roles during DVT progression, which may be an interesting topic worth further research. Furthermore, our data demonstrate that Tim-4 expression in macrophages of thrombus tissue is significantly elevated compared to other cell types within the lesion, indicating that macrophage-specific Tim-4 dysregulation primarily contributes to thrombotic pathogenesis rather than Tim-4 expressed in other cells.

Accumulating evidence has firmly established the pivotal role of inflammatory responses in DVT pathogenesis (45). Consistent with this, high-throughput full transcriptome sequencing profiling further identifies NF-κB signaling (the classical pathway of inflammatory signaling) as the most prominently enriched inflammatory pathway in DVT patients. In addition, transcriptomic analysis reveals significant upregulation of key inflammatory cytokines, including IL-1β, TNF-α and IL-6. Nevertheless, subsequent validation studies fail to demonstrate consistent upregulation of IL-6 (data not shown). Therefore, based on these findings, we only focus our subsequent analyses on inflammatory factors IL-1β and TNF-α as the most reliably upregulated inflammatory mediators in our experimental system.

Studies have demonstrated that NF-κB signaling activates more than 500 target genes involved in inflammatory responses and coagulation processes (46, 47). Our *in vitro* and *in vivo* experiments consistently demonstrate significant upregulation of macrophage-derived inflammatory cytokines (IL-1β and TNF-α) and increased phosphorylation of P65 and IκBα in the DVT microenvironment, strongly implicating NF-κB activation in thrombus pathogenesis. Subsequently, we employ pharmacological inhibition using BAY11-7082 to investigate whether Tim-4 contributes to DVT progression through NF-κB signaling. While NF-κB blockade substantially attenuated the pro-thrombotic phenotype observed in Tim-4 knockout mice, residual thrombus formation remained significantly elevated compared to inhibitor-treated wild-type controls. These results indicate that NF-κB signaling plays a central, but not exclusive, role in Tim-4-mediated DVT progression and Tim-4 may be involved in the progression of DVT in part through other signaling pathways, which warrants further exploration. The findings parallel observations in metabolic contexts that Tim-4 deficiency similarly exacerbates inflammation through NF-κB-mediated M1 macrophage polarization in obesity models (28), further supporting the conserved role of Tim-4 in regulating inflammation and positioning Tim-4 as a multimodal regulator of inflammatory thrombosis.

To elucidate how the transmembrane protein Tim-4 modulates NF-κB signaling activation, we integrated existing interactions with our findings. While Tim-4 has been shown to exert anti-inflammatory effects through LKB1-AMPKα-mediated autophagy activation (27), and TLR4 internalization-dependent NF-κB inhibition (28), its potential interaction with adaptor proteins in DVT remained unexplored. Notably, the protein kinase CK2 is known to directly activate NF-κB through phosphorylation of both P65 and IκBα. However, the potential involvement of CK2 in DVT pathogenesis, particularly its relationship with Tim-4, has not been previously investigated. Our study provides novel mechanistic insights by identifying CK2β as a direct binding partner of Tim-4 through Co-IP and LC-MS/MS analysis. The therapeutic potential of targeting this interaction was demonstrated by CX-4945 (a specific CK2 inhibitor), which markedly attenuated macrophage inflammatory responses and reduced endothelial prothrombotic factor expression, ultimately inhibiting DVT formation. These results extend recent discoveries in oncology, where TNF-α-induced protein 1 has been shown to suppress hepatocellular carcinoma progression by specifically blocking CK2β-mediated NF-κB activation (48). Nevertheless, limitations remained that we did not further identify the specific structural basis and key amino acids of Tim-4 binding to CK2β and whether additional adaptor proteins participate in this regulatory network.

Accumulating evidence indicates that Tim-4 expression is modulated by diverse inflammatory stimuli, including LPS, ConA, and various cytokines (49, 50). While the DVT microenvironment is similarly enriched with inflammatory mediators, their collective impacts on Tim-4 regulation remains unexplored. Notably, epigenetic regulation of Tim-4 has been demonstrated in dendritic cells from allergic mice, where P300-mediated chromatin remodeling at the Tim-4 promoter locus lead to its reduced expression in aged individuals (51, 52). However, whether analogous mechanisms function in DVT pathogenesis requires further investigation. Besides, emerging studies highlight the role of ncRNAs in regulation of Tim-4 abnormal expression from the perspective of epigenetic regulation. For instance, the upregulation of lncRNA NEAT1 enhances Tim-4 expression by sponging miR-202-3p (40). Our study extends this paradigm by identifying a novel ceRNA network in DVT: lncRNF219-3:1 overexpression upregulates Tim-4 expression by competitively binding miR-93-5p. The functional repertoire of miR-93-5p spans both oncogenic and protective roles across various pathologies. In cancer biology, miR-93-5p promotes tumor progression through: STAT3 pathway activation in gastric cancer (53), Cyclin-D2 targeting in ovarian cancer (54), and MAP3K2/c-Jun positive feedback loop in hepatocellular carcinoma (55). Conversely, miR-93-5p exhibits protective effects by serving as a prognostic biomarker in chronic thromboembolic pulmonary hypertension (56), and mitigating myocardial injury through Atg7/TLR4 inhibition (57) in progression of cardiovascular diseases. Our studies provide the first evidence of lncRNF219-3:1/miR-93-5p-mediated Tim-4 regulation in DVT, expanding the understanding of epigenetic control in thrombotic disorders, and positioning the lncRNF219-3:1/miR-93-5p/Tim-4 axis as a potential therapeutic target.

In recent years, lncRNAs functioning as ceRNAs have emerged as crucial post-transcriptional regulators in various disease processes, particularly cardiovascular disorders (58, 59). These ceRNAs modulate gene expression through miRNA sponging mechanisms, creating intricate networks that influence disease pathogenesis (60, 61). Our findings significantly expand this conceptual framework by identifying lncRNF219-3:1 as a novel ceRNA that orchestrates macrophage-mediated inflammatory responses and thrombotic progression *via* the miR-93-5p/Tim-4 regulatory axis. This mechanistic insight substantially enriches our understanding of the complex RNA-RNA interaction networks governing vascular pathophysiology. *In vitro* studies demonstrate that the lncRNF219-3:1/miR-93-5p axis regulates Tim-4 to orchestrate macrophage-mediated inflammatory responses and thrombotic progression in the THP-1 and HUVECs co-culture system. However, the *in vivo* functional relevance of this regulatory axis requires careful evaluation given the added complexity of hemodynamic forces, multicellular crosstalk, and systemic inflammatory cascades. Notably, significant discrepancies between *in vitro* and *in vivo* miRNA effects have been characterized. For instance, while miR-126 overexpression promotes thrombus resolution *in vitro* by stimulating EPCs proliferation, homing, migration, and angiogenic capacity (62), hypoxia-induced miR-126 downregulation *in vivo* impairs neovascularization *via* VEGF and MMP-9 suppression (63). These findings underscore that pathophysiological microenvironments *in vivo* such as hypoxia can significantly modify stability and function of miRNAs observed *in vitro*. Similarly, although static co-culture of ECs and smooth muscle cells transiently upregulates anti-inflammatory miRNAs (miR-146a, -708, -451, -98) in ECs, *in vivo* studies reveal sustained expression of these miRNAs under physiological shear stress, with marked suppression in regions of flow stagnation (64), underscoring the critical influence of shear stress variations on miRNA regulation. Furthermore, while miR-146a deficiency exacerbates thrombosis in mice, it does not affect neutrophil phagocytic capacity *in vitro* (65), underscoring the context-dependent roles of miRNA functions across experimental systems. Translation of these findings is further complicated by the poor evolutionary conservation of lncRNF219-3:1 across species. Unlike protein-coding genes, many lncRNAs exhibit limited homology between humans and experimental mammalian animals, presenting significant challenges for cross-species validation (66). To overcome these limitations, alternative approaches such as humanized xenograft models or patient-derived organoids may provide more physiologically relevant platforms for the translational potential of these findings (67, 68). Collectively, these findings imply that the lncRNF219-3:1/miR-93-5p axis may exert physiologically significant yet context-dependent roles *in vivo*, positioning it as a promising therapeutic target for vascular disorders.

## 5 Conclusion

In summary, our study identifies macrophage Tim-4 as a critical negative regulator that inhibits DVT development through selectively hijacking CK2β. Mechanistically, we demonstrate that macrophage specific *Tim-4* deficiency disrupts its binding to CK2β, thereby unleashing CK2-dependent activation of the NF-κB signaling pathway and elevating downstream proinflammatory genes expression, and eventually aggravating macrophage inflammatory responses-induced DVT progression. Furthermore, we uncover a novel regulatory axis governing Tim-4 downregulated expression in DVT: the ceRNA network mediated by lncRNF219-3:1/miR-93-5p axis. Based on these findings, we delineate a novel signaling pathway of Tim-4/CK2β/NF-κB in macrophage, modulated by lncRNF219-3:1/miR-93-5p axis. The findings not only provide new insights into the molecular pathogenesis of DVT but also highlight potential therapeutic targets for clinical DVT treatment.

## Data Availability

The datasets presented in this study can be found in online repositories. The names of the repository/repositories and accession number(s) can be found in the article/**Supplementary Material**.
