# Supplementary material for: Macrophage Tim-4 protects against deep vein thrombosis by binding CK2β to suppress inflammatory responses
Source: Front Immunol. 2025 Sep 23;16:1634230. doi: 10.3389/fimmu.2025.1634230 (PMC12500436; doi:10.3389/fimmu.2025.1634230)
Supplement: Supplementary file 13 [file Table2.docx]

Supplementary Material

# Supplementary Figures


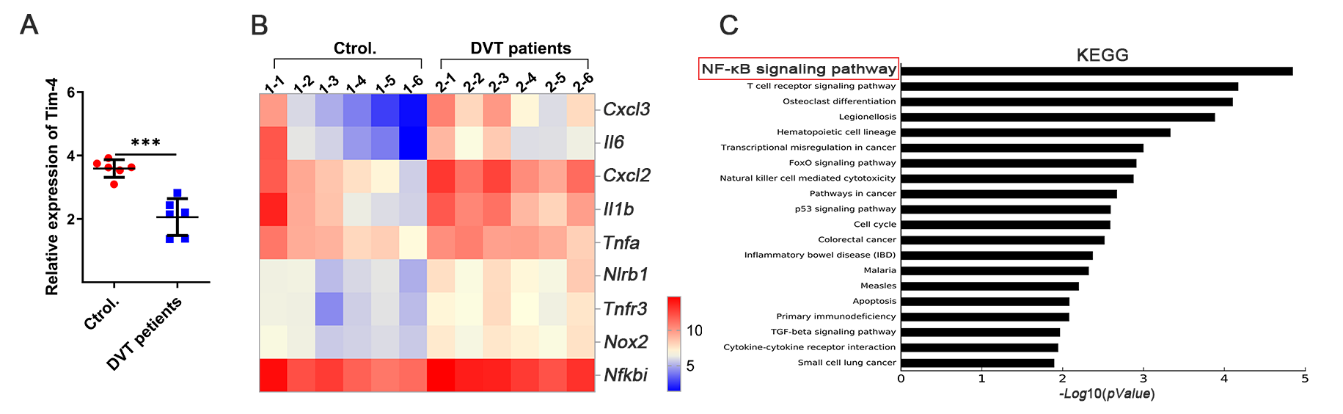


**Supplementary Figure 1. Tim-4 was down-regulated and pro-inflammatory factors were up-regulated in PBMCs of DVT patients from high-throughput whole transcriptome sequencing results, related to Figure 1.**

**(A)** The mRNA expression level of Tim-4 in PBMCs from DVT patients (n = 6) and controls (n = 6). **(B-C)** The heat map of related pro-inflammatory cytokines (left) and the top 20 KEGG-enriched signal pathways (right) from PBMCs of DVT patients (n = 6) and controls (n = 6) in microarray analysis. Error bars indicate SD of at least three biological replicates per group in one experiment. *** *p* < 0.001.


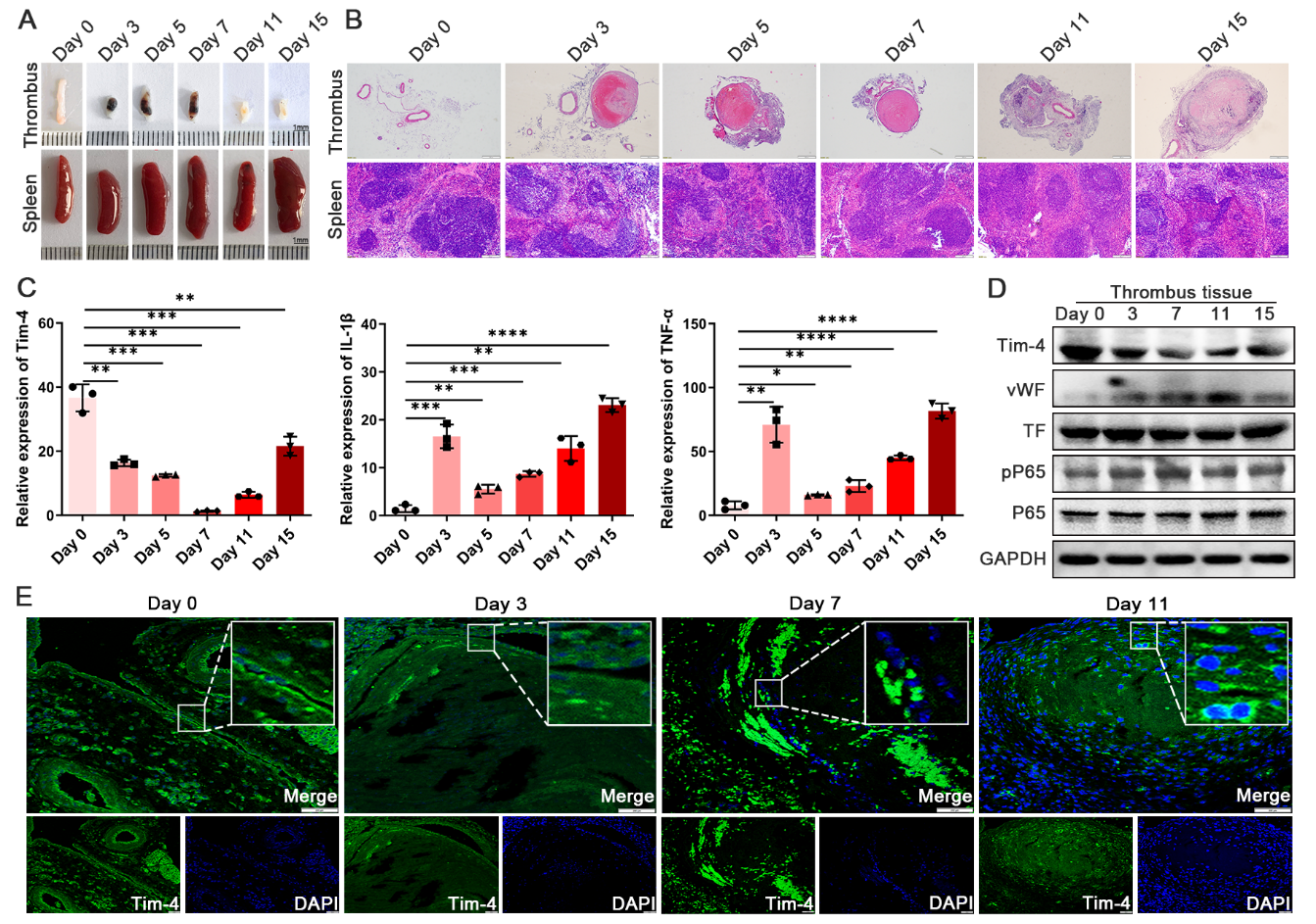


**Supplementary Figure 2. Tim-4 decreased first and then increased in IVCs of DVT mice, related to Figure 1.**

**(A)** Representative images of IVCs thrombosis and spleen from DVT mice on days 0, 3, 5, 7, 11, and 15. Scale bar, 1 mm. **(B)** Representative microscopic images of HE staining in cross-sections of the thrombus (scale bar, 500 μm) and spleen (scale bar, 200 μm) from DVT mice on days 0, 3, 5, 7, 11, and 15. **(C)** qPCR analysis for Tim-4, IL-1β, and TNF-α in thrombus tissue from DVT mice on days 0, 3, 5, 7, 11, and 15, respectively. **(D)** Western blot analysis of Tim-4, vWF, TF, pP65, and P65 expression in thrombus tissue from DVT mice on days 0, 3, 7, 11, and 15, respectively. **(E)** Representative IF photomicrographs of thrombus sections with Tim-4 Ab (green) in DVT mice. Nuclei were stained with DAPI (blue). Scale bar, 200 μm. Error bars indicate SD of at least three biological replicates per group in one experiment. * *p* < 0.05, ** *p* < 0.01, *** *p* < 0.001, **** *p* < 0.0001.


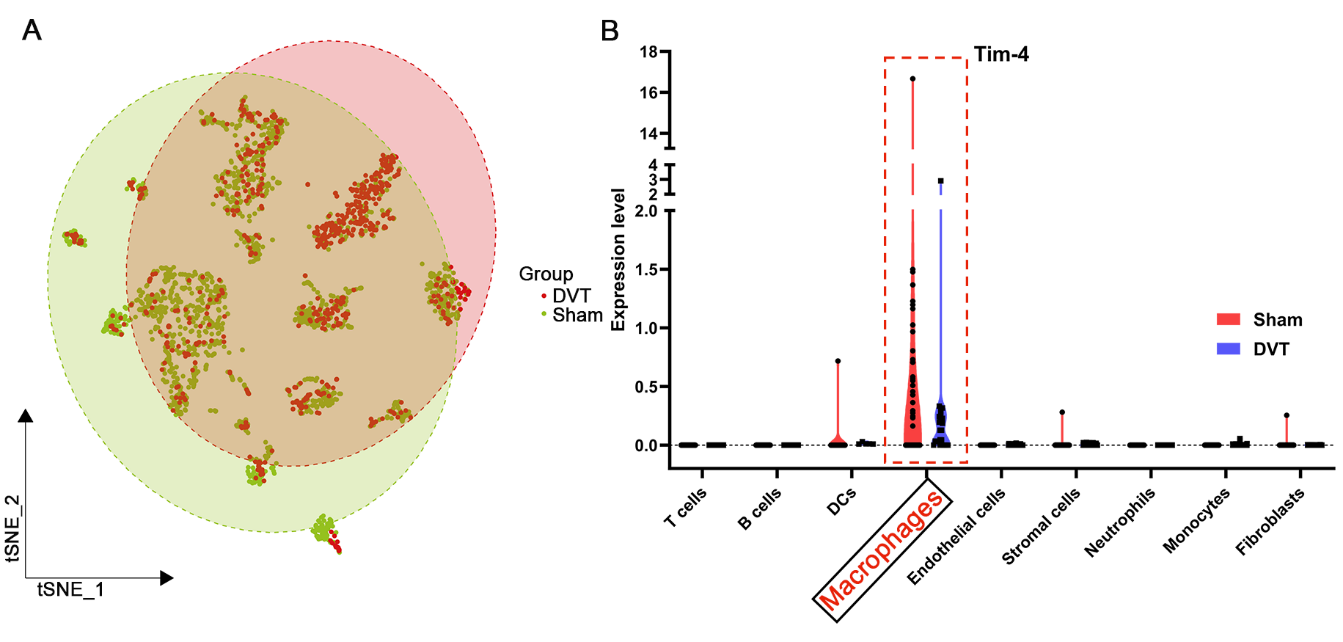


**Supplementary Figure 3. The differential expression of Tim-4 was analyzed by single cell sequencing, related to Figure 1.**

**(A)** T-distributed stochastic neighbor embedding (t-SNE) plot of different cell clusters from murine inferior vena cavas (IVCs, Sham and DVT data combined). **(B)** Violin diagram of Tim-4 expression in different cell clusters from sham and DVT mice.


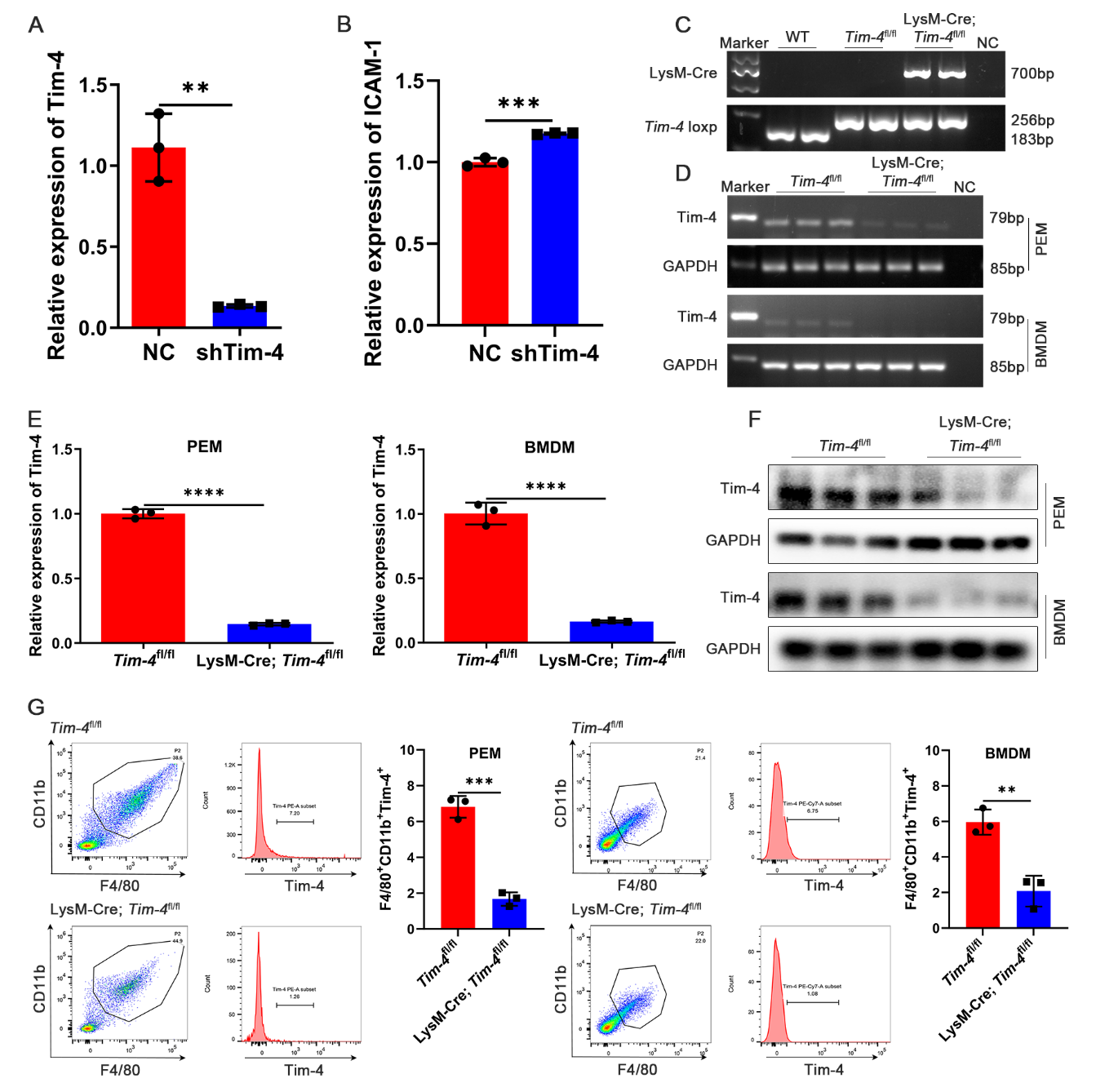


**Supplementary Figure 4. Knockout efficiency of Tim-4 in LysM-Cre; *Tim-4*^fl/fl^ mice and *Tim-4*^fl/fl^ mice, related to Figure 2.**

**(A)** Relative mRNA level of Tim-4 in THP1 after transfection with LV-shTim-4 or LV-NC for 72 hours. **(B)** Relative mRNA level of ICAM-1 in HUVEC co-cultured with THP1 transfected with LV-shTim-4 or LV-NC. **(C)** PCR for genotyping used genomic DNA from the mouse toe. **(D-G)** Knockout efficiency was assessed in PEM and BMDM from Tim-4^fl/fl^ and LysM-Cre; *Tim-4*^fl/fl^ mice using regular PCR, qPCR, WB, and FCM analysis. Error bars indicate SD of at least three biological replicates per group in one experiment. ** *p* < 0.01, *** *p* < 0.001, **** *p* < 0.0001.


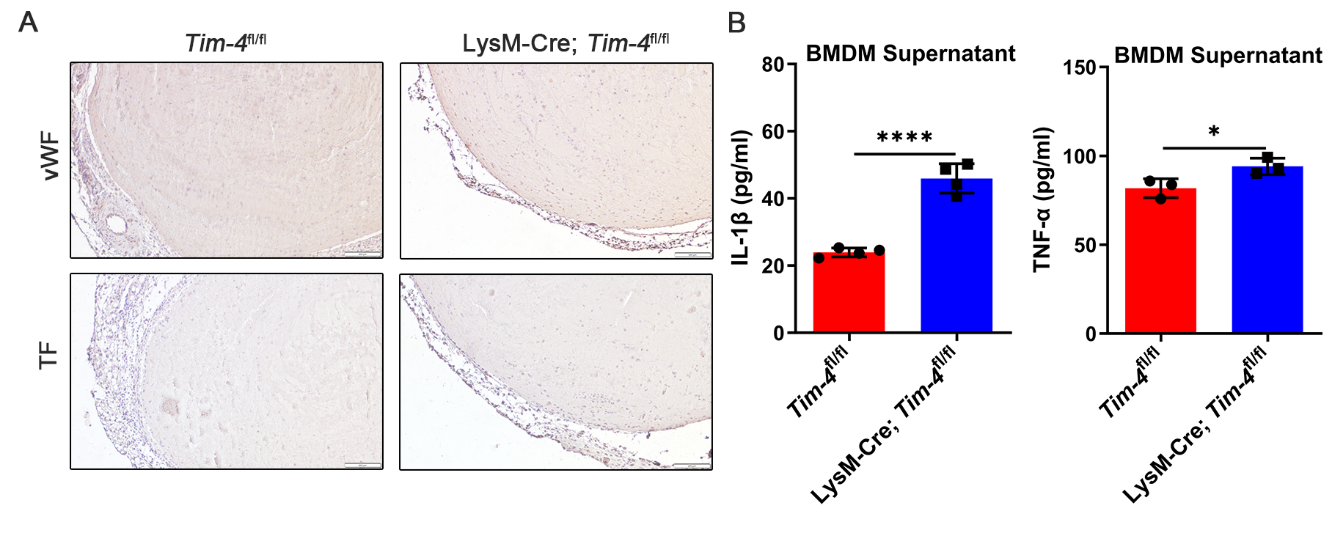


**Supplementary Figure 5. The absence of *Tim-4* in macrophages promotes thrombosis formation, related to Figure 2.**

**(A)** Representative IHC staining of thrombus tissues with vWF and TF Ab in Tim-4^fl/fl^ and LysM-Cre; *Tim-4*^fl/fl^ mice. Scale bar, 200 μm. **(B)** ELISA for IL-1β and TNF-α in BMDM culture medium from Tim-4^fl/fl^ and LysM-Cre; *Tim-4*^fl/fl^ mice. Error bars indicate SD of at least three biological replicates per group in one experiment. * *p* < 0.05, **** *p* < 0.0001.


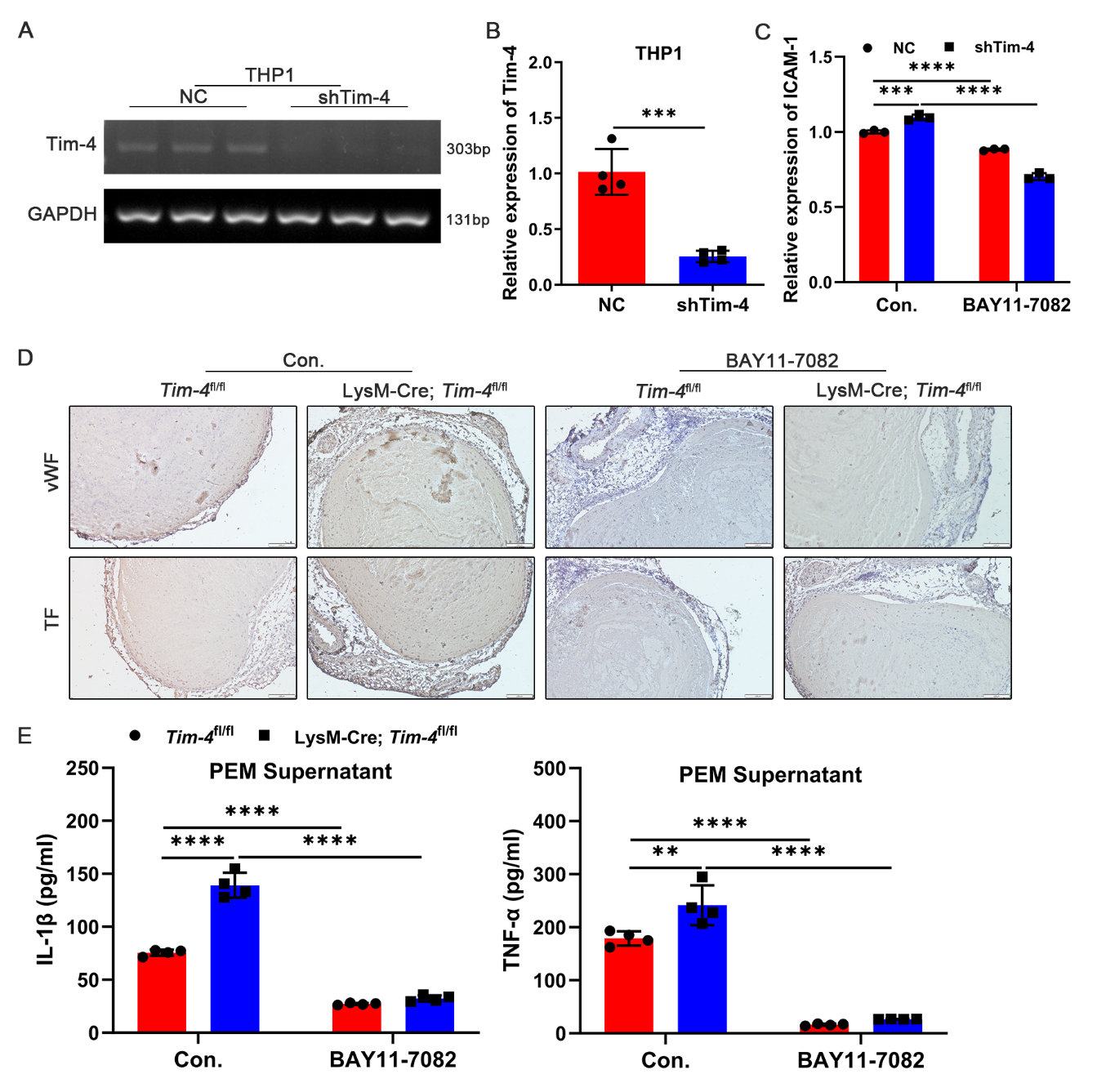


**Supplementary Figure 6. Macrophage Tim-4 inhibits DVT formation through the NF-κB signaling pathway, related to Figure 3.**

**(A-B)** Relative mRNA level of Tim-4 in THP1 after transfection with LV-shTim-4 or LV-NC for 72 hours was detected by regular PCR and qPCR. **(C)** THP1 was transfected with LV-shTim-4 or LV-NC for 72 hours and treated with LPS for 0.5 hour, and inhibitor groups were treated with BAY11-7082 for another 1 hour and co-cultured with HUVEC. Relative mRNA level of ICAM-1 in HUVEC co-cultured with THP1. **(D)** Representative IHC staining of thrombus tissues with vWF and TF Ab in Tim-4^fl/fl^ and LysM-Cre; Tim-4^fl/fl^ mice with or without BAY11-7082 treatment. Scale bar, 200 μm. **(E)** ELISA for IL-1β and TNF-α in PEM culture medium from Tim-4^fl/fl^ and LysM-Cre; Tim-4^fl/fl^ mice with or without BAY11-7082 treatment. Error bars indicate SD of at least three biological replicates per group in one experiment. ** *p* < 0.01, *** *p* < 0.001, **** *p* < 0.0001.


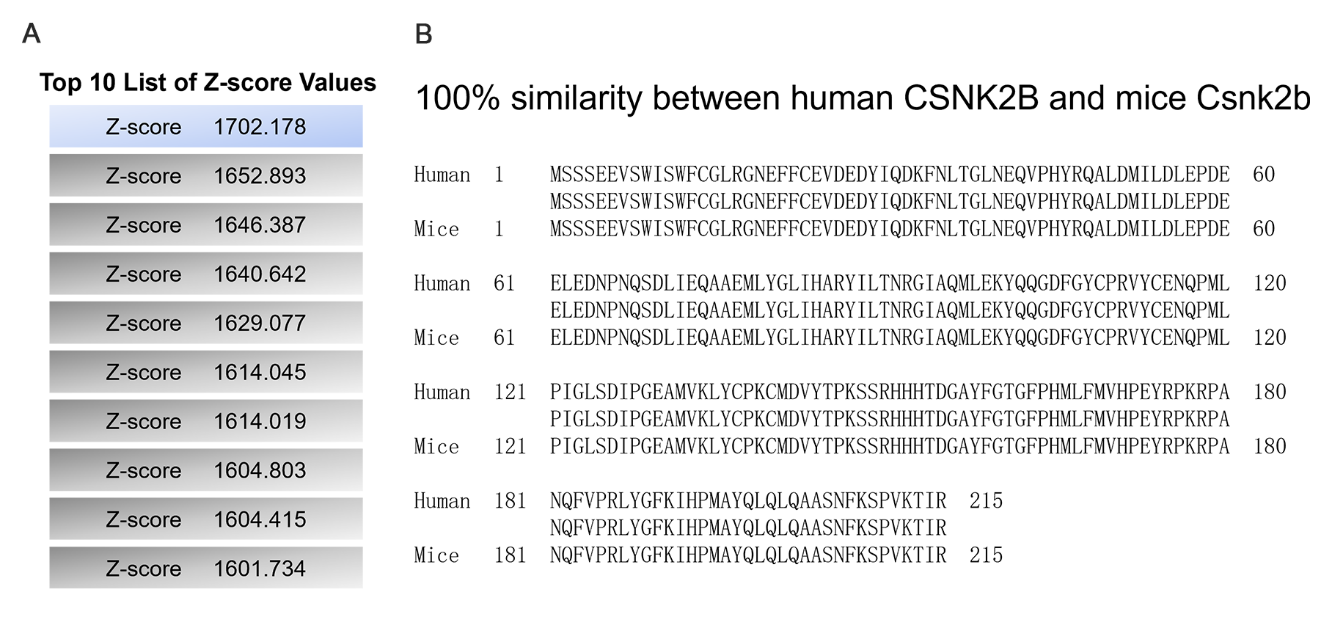


**Supplementary Figure 7. Screening Tim-4 binding protein, related to Figure 4.**

**(A)** The top 10 list of Z-score values of Tim-4 and CK2β. **(B)** CK2β protein sequence homology alignment of human and mouse.


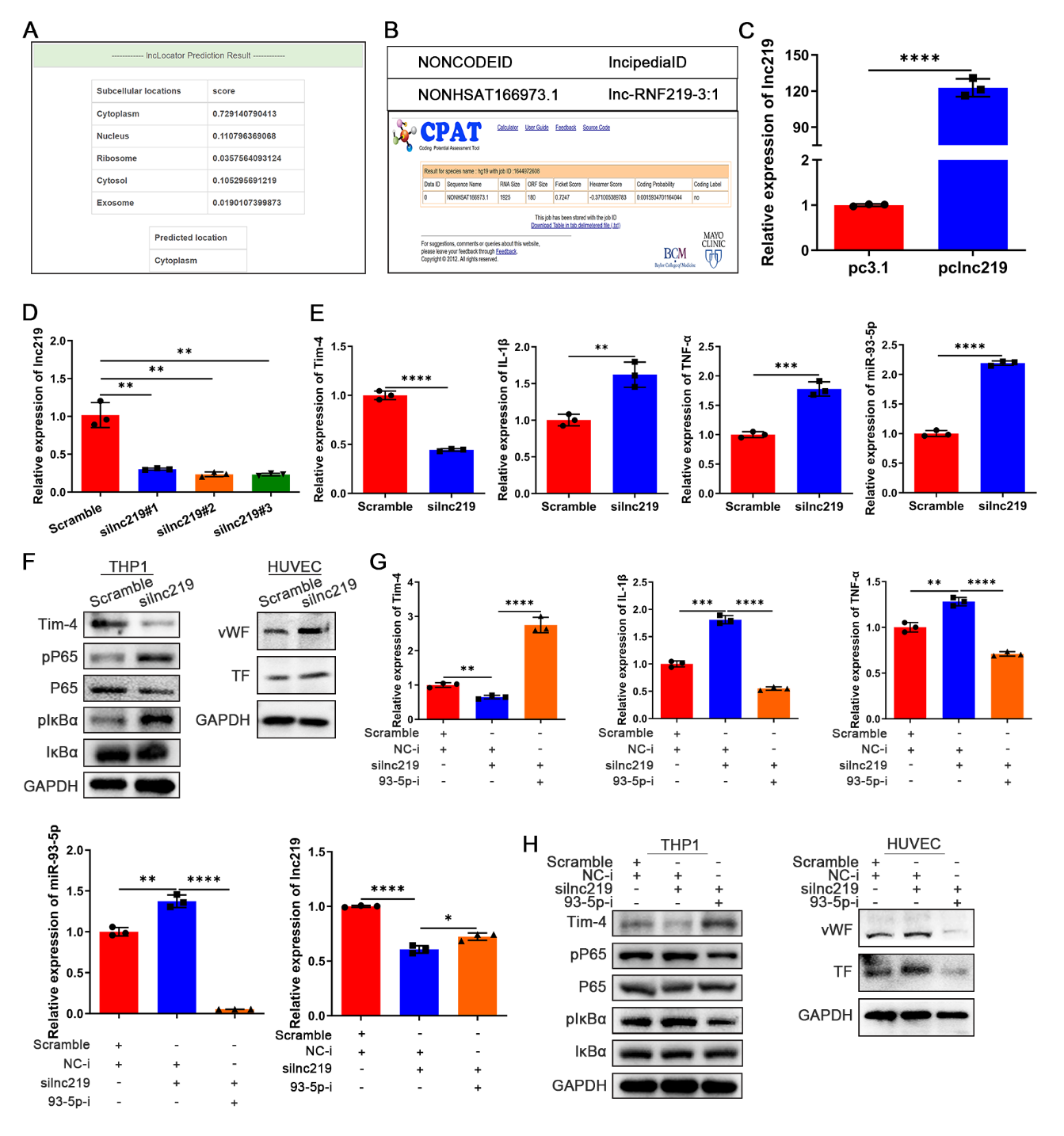


**Supplementary Figure 8. Screening the target lncRNA-lnc219 of miR-93-5p, related to Figure 6.**

**(A)** The subcellular locations of lnc219 were analyzed in THP1. **(B)** The coding potential assessment tool was used to predict the coding ability of lnc219. **(C)** THP1 was transfected with pclnc219 or pcDNA3.1 for 48 hours. The expression of lnc219 was evaluated by qPCR. **(D)** qPCR analysis for lnc219 in THP1 transfected with silnc219#1, silnc219#2, silnc219#3, or scramble for 48 hours, respectively. **(E)** qPCR analysis for Tim-4, IL-1β, TNF-α, and miR-93-5p in THP1 transfected with silnc219 or scramble for 48 hours, respectively. **(F)** Western blot analysis of Tim-4, pP65, P65, pIκBα, and IκBα expression in THP1 transfected with silnc219 or scramble for 48 hours and western blot analysis of vWF and TF in HUVEC co-cultured with THP1, respectively. **(G-H)** THP1 was transfected with silnc219 or scramble for 48 hours and then transduced with miR-93-5p inhibitor or negative control inhibitor for 48 hours, followed by co-cultivation with HUVEC. **(G)** qPCR analysis for Tim-4, IL-1β, TNF-α, miR-93-5p, and lnc219 in THP1 described as above. **(H)** Western blot analysis of Tim-4, pP65, P65, pIκBα, and IκBα expression in THP1 and western blot analysis of vWF and TF in HUVEC co-cultured with THP1 described as above. Error bars indicate SD of at least three biological replicates per group in one experiment. NC-i, negative control inhibitor, 93-5p-i, miR-93-5p inhibitor. * *p* < 0.05, ** *p* < 0.01, *** *p* < 0.001, **** *p* < 0.0001.

# Supplemental Materials and methods

## Genotyping

One step mouse genotyping kit (PD101-01, Vazyme, Nanjing, China) was used to extract DNA from 1-2-week-old mice after cutting their toes. Briefly, the tissue was immersed in a lysate pre-added with proteinase K, incubated at 55 °C for 20 minutes, and then heated at 95 °C for 5 minutes to inactivate proteinase K. The lysate could be directly used as a PCR amplification template after centrifugation. Finally, the amplified products were directly detected by agarose gel electrophoresis.

- 1. **Preparation of conditioned medium**

HUVEC were cultured in serum-free DMEM/F-12 (1:1) medium for 24 hours, and the culture supernatant was collected. Then 300 μl of culture supernatant or serum from DVT patients was prepared and added into the complete medium to reach the final volume of 1000 μl as conditioned medium, which was used to stimulate THP1 cells for inducing DVT microenvironments.

- 1. **Lentiviruses infected THP1 cells**

Briefly, THP1 were seeded at 2×10^5^ cells/ml on 12-well plates. Lentivirus-mediated short hairpin RNA targeting Tim-4 (Lv-shTim-4) and Lentivirus-negative control (Lv-NC) were transfected into THP1 (MOI = 20) respectively. The volume of culture medium without FBS was 0.5 ml/well in a 12-well plate, and 1 × HitransGA was added to enhance the infection efficiency. After 12 hours, the medium was replaced with fresh culture medium containing 10% FBS to maintain cell viability.

- 1. **Cells co-culture system**

THP1 cells were treated with 100 ng/ml phorbol 12-myristate 13-acetate (PMA, S7791, Selleck, Houston, USA) for 24 hours to adhere to the wall and then cultured in RPIM-1640 medium containing 10% FBS and 1 μg/ml LPS for 30 minutes. Then, the inhibitor group was treated with 10 μM BAY11-7082 or 50 μM CX-4945 for 1 hour. Co-culture experiments used 24-well transwell plates with 0.4 µm pore polycarbonate membrane inserts (3413, Corning Costar, USA). With THP1 planted in the bottom chamber, HUVEC were seeded in transwell inserts. Finally, HUVEC and THP1 cells were collected respectively for subsequent experiments.

- 1. **Hematoxylin-eosin (HE) and Immunohistochemistry (IHC) Staining**

Freshly-obtained mouse thrombus and spleen samples were fixed in 4% paraformaldehyde (BL539A, Biosharp, Hefei, China) and embedded in paraffin, followed by sectioning. For HE staining, the tissue sections were stained with hematoxylin (G1120, Solarbio, Beijing, China) for 3 minutes and eosin solution (G1120, Solarbio, Beijing, China) for 3 minutes. For IHC staining, antigen retrieval, endogenous peroxidase blocking, and washing procedures were identical to those used in IF staning. The distinctions were primary antibodies against TF (CY5807, Abways, Shanghai, China) and vWF (11778-1-AP, Proteintech, Wuhan, China), and corresponding secondary antibodies (SP-9000, ZSGB-BIO, Beijing, China). Finally, the nucleus was counterstained with hematoxylin (H8070, Solarbio, Beijing, China). Subsequently, images were acquired using a conventional inverted fluorescence microscope (Olympus).

- 1. **Enzyme-linked immunosorbent assay (ELISA)**

The culture supernatants of mouse PEMs or BMDMs were collected and centrifuged at 500 g for 5 minutes. Subsequently, the levels of IL-1β and TNF-α were detected by corresponding double antibody sandwich ELISA detection kit (KE10003, Proteintech, Wuhan, China; KE10002, Proteintech, Wuhan, China). All experimental steps were performed according to the instructions. With 630 nm as the calibration wavelength, the optical density of each hole was measured at 450 nm using a full-wavelength microplate reader. Create a standard curve using the standard in the kits. ELISACalc software was used for data analysis.

- 1. **Bioinformatics prediction and luciferase reporter assay**

The potential binding sites between miR-93-5p and Tim-4, or lncRNF219-3:1, were predicted by several online databases, including TargetScan, miRDB, miRWalk, NONCODE etc. LncRNF219-3:1 and Tim-4 3'UTR sequences containing putative miR-93-5p binding sites were amplified and then cloned into a pmirGLO vector, named lncRNF219-3:1-WT and Tim-4-WT. The putative miR-93-5p binding site was mutated to generate corresponding mutant plasmids (lncRNF219-3:1-MUT and Tim-4-MUT). HEK293T cells were inoculated in 96-well plates in advance. To verify the direct binding of miR-93-5p to the Tim-4 mRNA 3'UTR, HEK293T cells were co-transfected with miR-93-5p mimics/mimic control and plasmids containing Tim-4-WT or Tim-4-MUT. Similarly, in order to determine the interaction between miR-93-5p and lncRNF219-3:1, miR-93-5p mimics/mimic control, and plasmids containing lncRNF219-3:1-WT or lncRNF219-3:1-MUT were introduced into HEK293T cells. After 48 hours of transfection, the activity of firefly and renilla luciferase was detected on a microplate luminometer using a commercial dual-luciferase reporter system.

- 1. **RNA fluorescence in situ hybridization (FISH)**

The location of lncRNF219-3:1 or miR-93-5p in THP1-derived macrophages was localized using the RNA FISH kit SA-Biotin system (B038-V003-20201215, Genepharma, Shanghai, China). Cells were hybridized with specific biotin-labeled lncRNF219-3:1 or miR-93-5p probes at 37 °C overnight, which was synthesized by Genepharma (Shanghai, China). Then the nucleus was stained with DAPI. Fluorescence images were observed and photographed using a fluorescence microscope.

- 1. **RNA Antisense Purification (RAP)**

The relationship between lncRNF219-3:1 and miR-93-5p was determined using an RAP kit (RAP bes5103, BersinBio, Guangzhou, China) and miRNA pull down kit (bes5108, BersinBio, Guangzhou, China) under manufacturer’s protocol. Briefly, for RAP assay, crossed-linked THP1 cells were collected, lysed, and hybridized with biotin-labeled lncRNF219-3:1 probe. Then lncRNF219-3:1 and its interacting RNAs were captured by streptavidin magnetic beads. After RNA elution and purification, reverse transcription RNA was measured by qPCR to obtain enrichment efficiency.

- 1. **miRNA pull down assay**

For miRNA pull down assay, biotin-labeled miR-93-5p (Bio-miR-93-5p) and biotin-labeled the negative control (Bio-NC) were introduced into THP1-derived macrophages, respectively. After 48 hours incubation, cells were collected and lysed. The lysates were incubated with streptavidin magnetic beads in a rotary shaker at 4 °C for 3 hours. At the same time, non-specific binding was blocked using RNase-free BSA and yeast tRNA. These beads were washed twice with the lysis buffer, three times with low-salt buffer, and once with high-salt buffer. Finally, the binding RNA was isolated by TRIzol reagent, and the expression of lncRNF219-3:1 was detected by qPCR.
