# Supplementary material for: Macrophage Tim-4 protects against deep vein thrombosis by binding CK2β to suppress inflammatory responses
Source: Front Immunol. 2025 Sep 23;16:1634230. doi: 10.3389/fimmu.2025.1634230 (PMC12500436; doi:10.3389/fimmu.2025.1634230)

## Slide 1
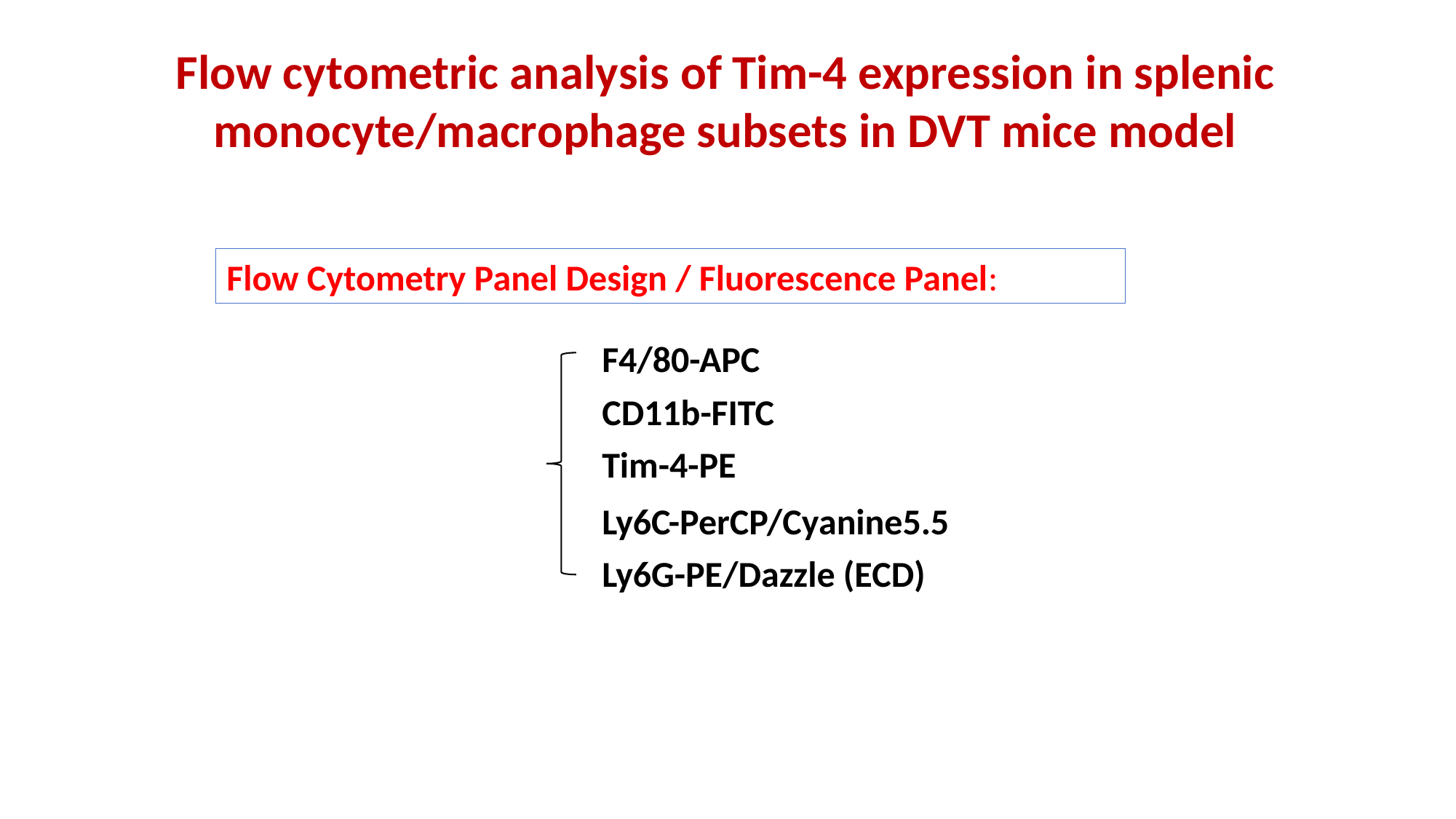

Flow cytometric analysis of Tim-4 expression in splenic monocyte/macrophage subsets in DVT mice model
Flow Cytometry Panel Design / Fluorescence Panel:
F4/80-APC
CD11b-FITC
Tim-4-PE
Ly6C-PerCP/Cyanine5.5
Ly6G-PE/Dazzle (ECD)

## Slide 2
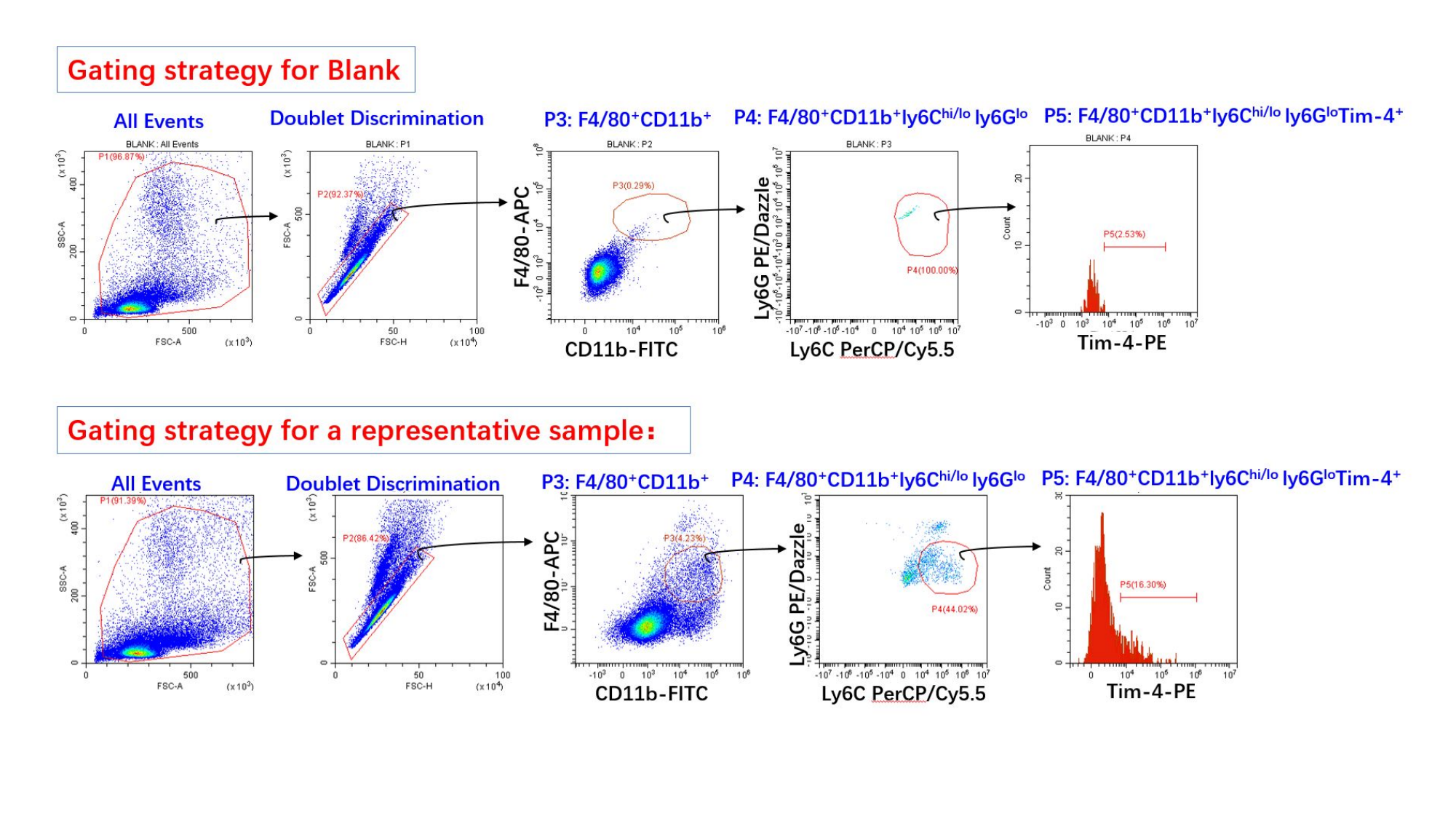

## Slide 3
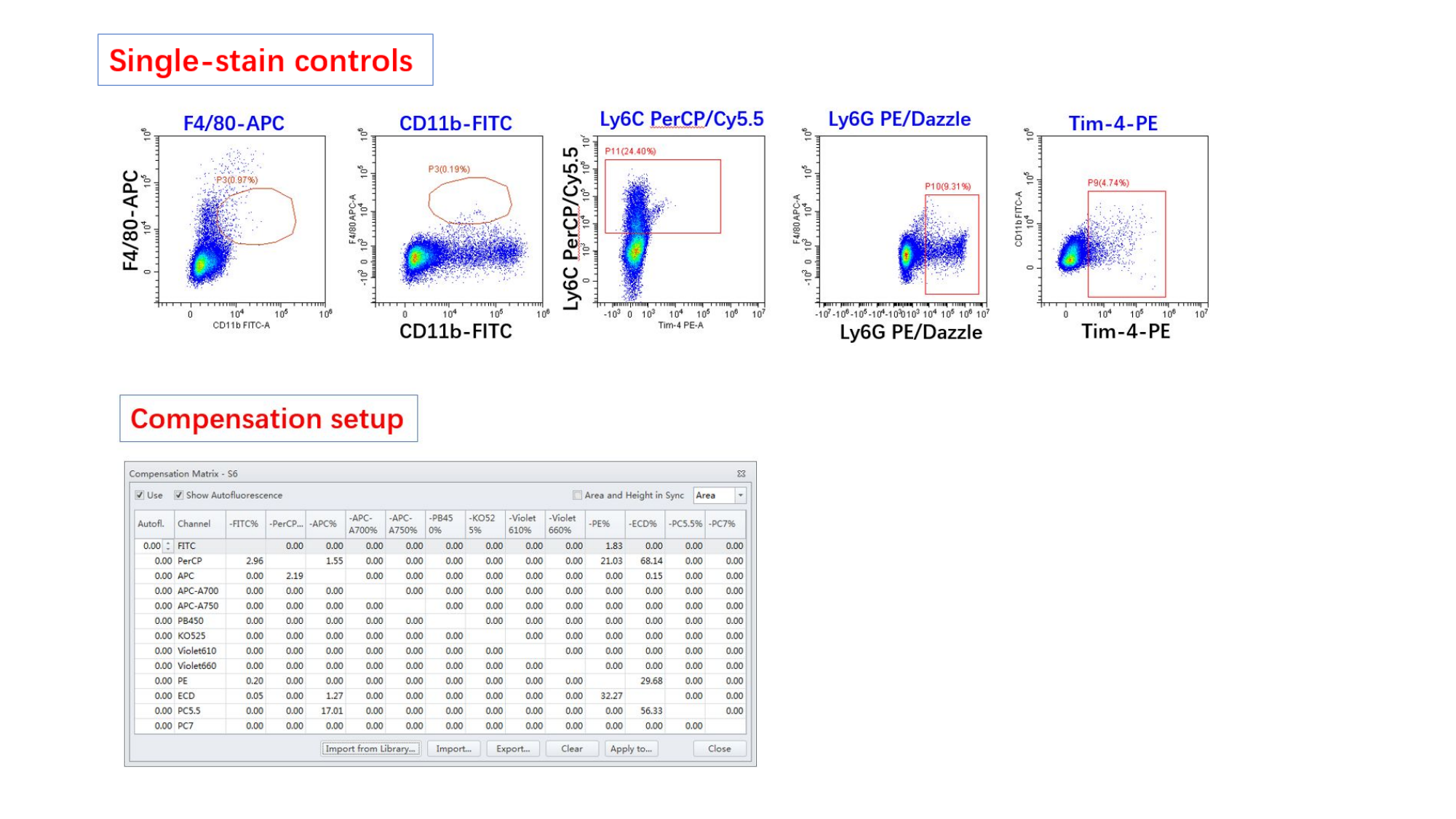

## Slide 4
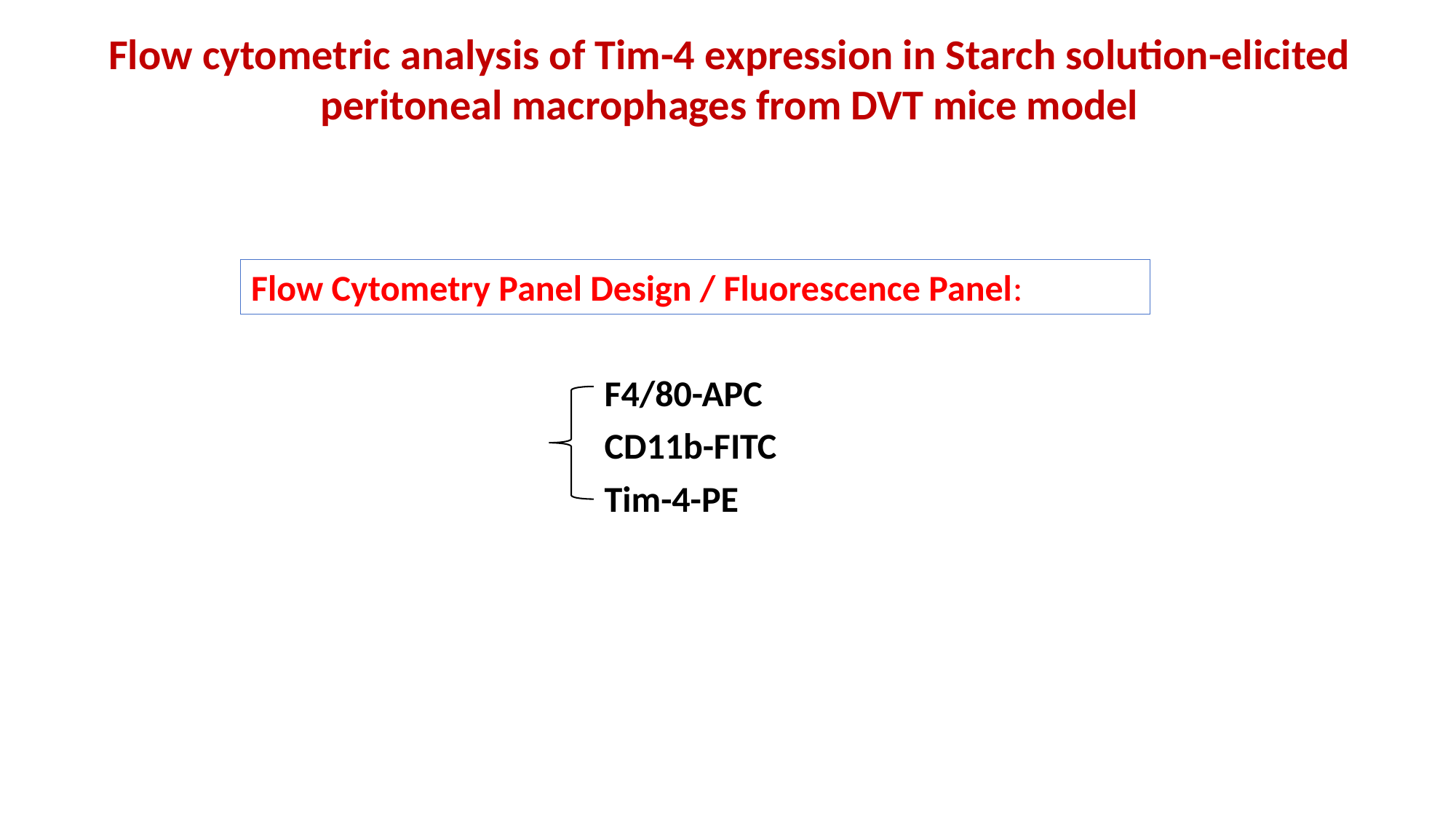

Flow cytometric analysis of Tim-4 expression in Starch solution-elicited peritoneal macrophages from DVT mice model
Flow Cytometry Panel Design / Fluorescence Panel:
F4/80-APC
CD11b-FITC
Tim-4-PE

## Slide 5
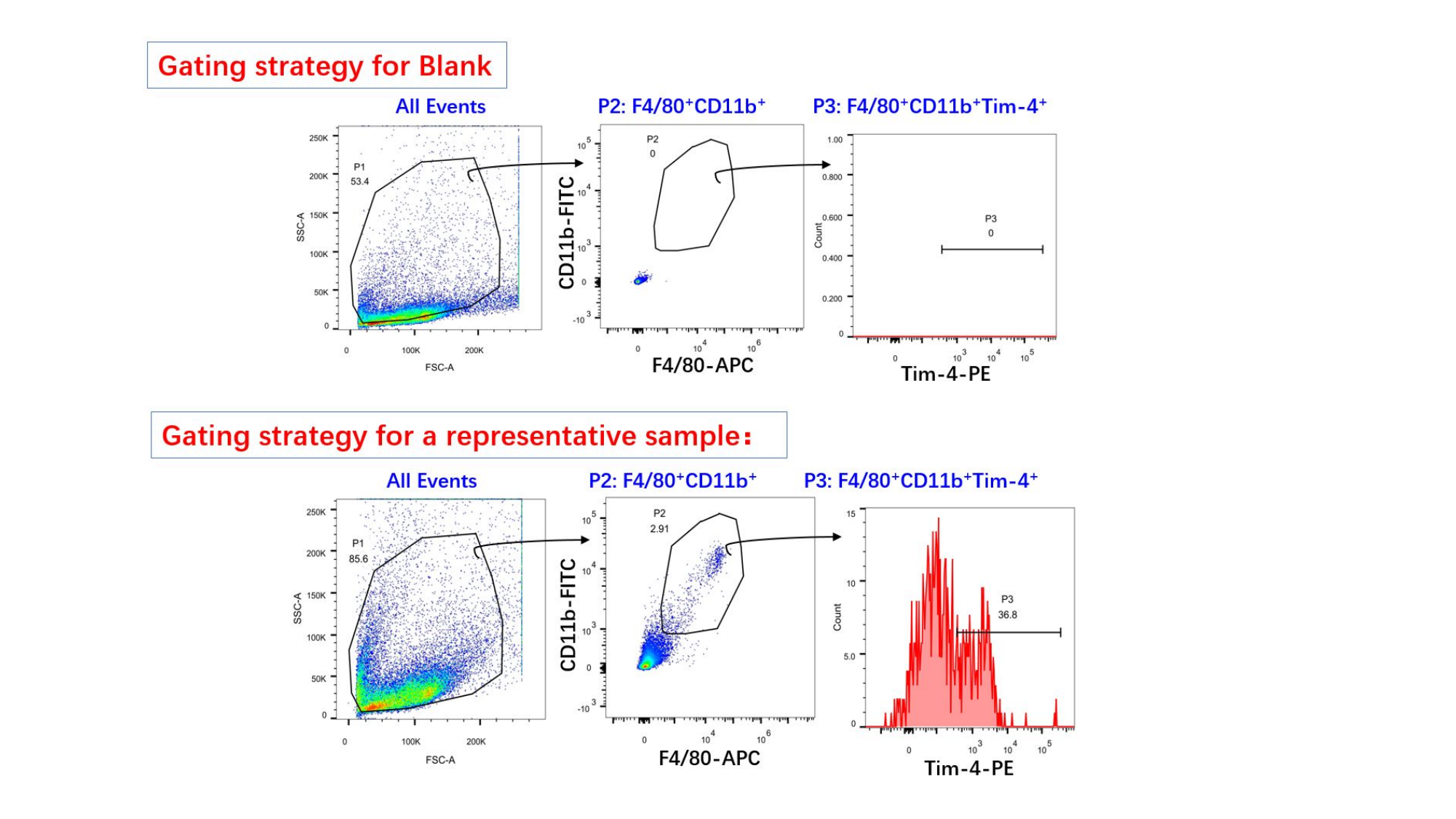

## Slide 6
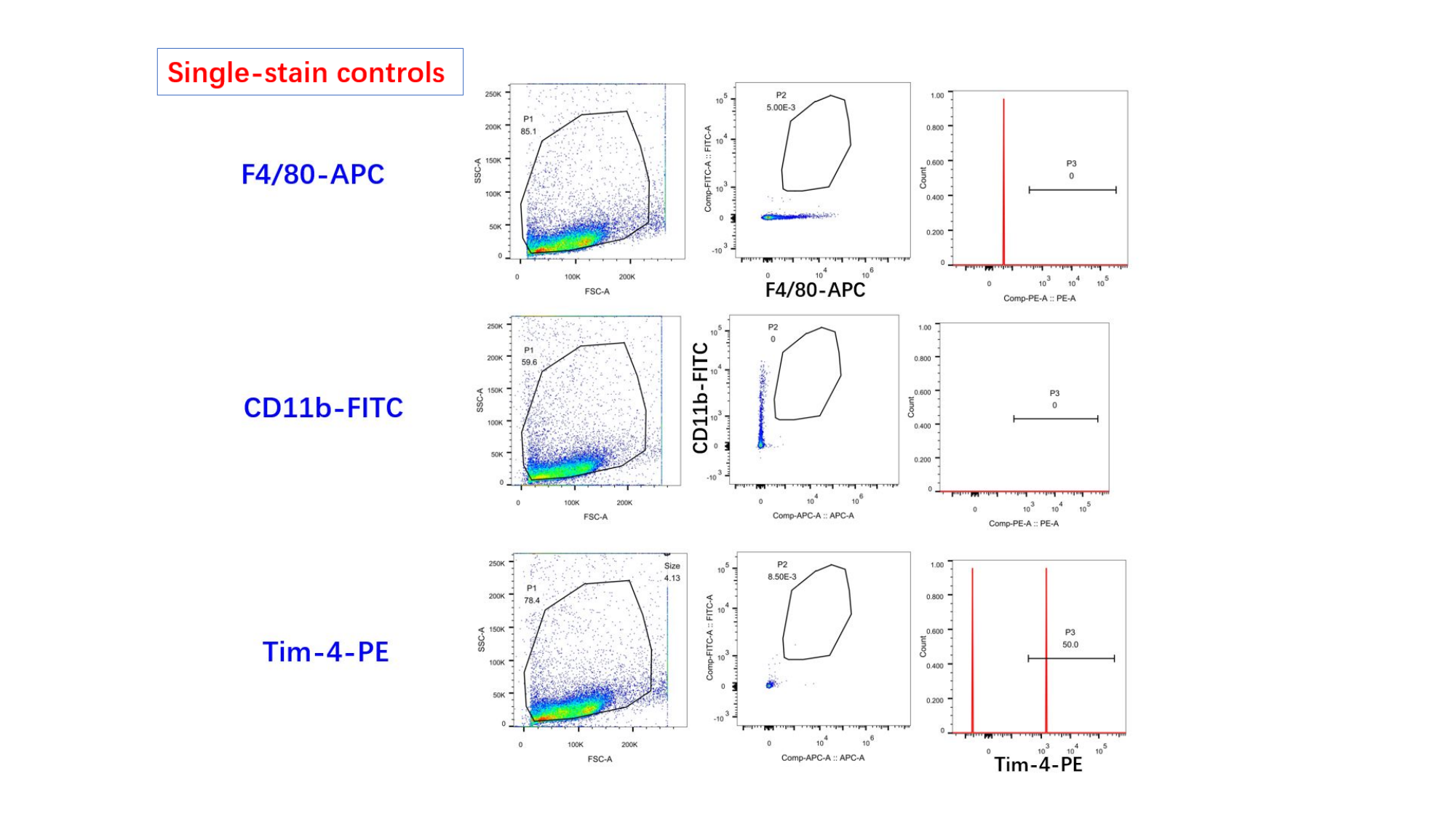

Supplement: Supplementary file 14 [file Presentation1.pptx]
